# Composition of lipid nanoparticles for targeted delivery: application to mRNA therapeutics

**DOI:** 10.3389/fphar.2024.1466337

**Published:** 2024-10-23

**Authors:** Olga Vasileva, Olga Zaborova, Bogdan Shmykov, Roman Ivanov, Vasiliy Reshetnikov

**Affiliations:** ^1^ Translational Medicine Research Center, Sirius University of Science and Technology, Sochi, Russia; ^2^ Chemistry Department, Moscow State University, Moscow, Russia

**Keywords:** lipid nanoparticles, mRNA therapeutic, *in vivo*, nucleic acid delivery system, drug development, gene editing

## Abstract

Today, lipid nanoparticles (LNPs) are some of the main delivery systems for mRNA-based therapeutics. The scope of LNP applications in terms of RNA is not limited to antiviral vaccines but encompasses anticancer drugs and therapeutics for genetic (including rare) diseases. Such widespread use implies high customizability of targeted delivery of LNPs to specific organs and tissues. This review addresses vector-free options for targeted delivery of LNPs, namely the influence of lipid composition of these nanoparticles on their biodistribution. In the review, experimental studies are examined that are focused on the biodistribution of mRNA or of the encoded protein after mRNA administration via LNPs in mammals. We also performed a comprehensive analysis of individual lipids’ functional groups that ensure biodistribution to desired organs. These data will allow us to outline prospects for further optimization of lipid compositions of nanoparticles for targeted delivery of mRNA therapeutics.

## 1 Components of lipid nanoparticles (LNPs)

During more than three decades of research developments, approaches to the fabrication of LNPs have undergone major changes. With the investigation of lipid-based delivery systems, the composition of the nanoparticles has become more complex: in addition to the cholesterol and phospholipid used in the first LNPs, new components have come onto the scene that have improved desired properties of the nanoparticles. All the LNPs approved to date for clinical use consist of four lipids: an ionizable cationic lipid, cholesterol, a phospholipid (helper lipid), and a polyethylene glycol (PEG)-conjugated lipid (PEGylated lipid). Due to this composition, it is possible to obtain a monodisperse system of LNPs that allow to encapsulate nucleic acids with high efficiency and are capable of efficiently delivering them into the cell (Kulkarni et al., 2019).

Among all the other components of LNPs, the cationic lipid constitutes the largest proportion: ∼50 mol% of the total lipids. Due to the large excess of a positive charge, there is a risk of an immune response, which can dramatically reduce delivery efficiency of LNPs. To attenuate the toxic effect, modern formulations of LNPs involve ionizable cationic lipids designed in such a way that at physiological pH (7.4), the lipid is in a neutral state (Sato, 2021). A structurally ionizable lipid is most often a tertiary amine that is deprotonated in a neutral environment and positively charged at pH below the acidity constant (pK_a_) of the lipid. Various structures of cationic lipids are known that differ in polar groups (mono- or polycation and linear or heterocyclic) and hydrophobic moieties (cholesterol-based or linear). In the practice of LNP preparation, linear monocationic lipids are used mostly. Ionizable cationic lipids perform key functions in lipid particle systems: first, they effectively bind to negatively charged nucleic-acid residues and encapsulate them into particles; second, they ensure the neutral charge of nanoparticles at physiological pH; and third, they improve endosomal fusion by destabilizing the endosomal membrane when the charge turns positive after endosome acidification (Cheng and Lee, 2016).

Cholesterol is the second most abundant lipid (∼40 mol%) in LNPs. It performs two main functions: first, it extends effective half-life of LNPs in the bloodstream by diminishing the amount of surface-bound proteins; and second, it ensures preservation of the encapsulated material and its delivery into the cell by altering the rigidity of the particle membrane (Hald et al., 2022).

The most commonly used phospholipid is 1,2-distearoyl-*sn*-glycero-3-phosphocholine (DSPC), which has a high melting point (55°C), which enables increased circulation time in the body and higher stability of nanoparticles. The addition of 1,2-dioleoyl-*sn*-glycero-3-phosphoethanolamine (DOPE) into nanoparticles improves intracellular delivery. The DOPE molecule is a truncated cone owing to the presence of a double bond in the *cis* configuration in each alkyl chain and of a small polar group, which allow for the formation of hexagonal layers. For this reason, during an endosomal release of RNA, the presence of DOPE within LNPs can accelerate the fusion of the two membranes, thus improving the efficiency of de-encapsulation of nucleic acids. The phospholipid content of modern LNPs is ∼10 mol% (Hald et al., 2022).

Another important component of LNPs that is relevant to their clinical use is the PEGylated lipid, whose proportion in LNPs is ∼1.5 mol%. PEGylated lipids are responsible for some key properties of LNPs (Hald et al., 2022; Evers et al., 2018): particle sizes and their distribution as well as resistance to aggregation. The PEGylated lipid also modulates the body’s immune response, determines the pharmacokinetics and pharmacodynamics of the nanoparticles, and affects the efficiency of nucleic-acid encapsulation.

The use of nonviral systems of delivery of nucleic acids by means of nanoparticles, especially LNPs, opens up unprecedented opportunities for their targeted delivery to desired organs. The classic four-component composition of LNPs (an ionizable cationic lipid, cholesterol, helper lipid, and PEGylated lipid) helps LNPs to get into most organs and tissues, with predominant localization to the liver. Several *in vivo* studies have shown that modifications of lipid composition help to “redirect” the nanoparticles to the lungs and spleen (Meyer et al., 2022; Dilliard et al., 2021; Gueguen et al., 2024). Pilot studies on rodents were recently published that present successful targeting of LNPs to various types of tumors, to the pancreas, placenta, brain, retina, and spermatocytes (Blakney et al., 2019; Xiong et al., 2020; Liu et al., 2022; Melamed et al., 2023; Young et al., 2022; Tuma et al., 2023; Waggoner et al., 2023; Patel et al., 2019; Ryals et al., 2020; Du et al., 2023). In addition to “targeting” of LNPs via the modifications of lipid composition, the incorporation of various protein macromolecules and antibodies into LNPs seems to be another promising field (Lee et al., 2023).

Active targeting of LNPs can be achieved via incorporation of presynthesized vectors (specific lipids or small-molecule compounds conjugated to lipids) into the nanoparticles or via postmodification of LNPs (attachment of antibodies) (Lin et al., 2023). Inclusion of a mannose moiety leads to active transport of the nanoparticles into dendritic cells, which is actively exploited to develop antitumor drugs (Lei et al., 2024) or specific vaccines, for example, against African swine fever (Gong et al., 2024), as well as to improve intradermal delivery of LNPs (Goswami et al., 2019). Another example of a small-molecule compound for active delivery is the use of a neurotransmitter conjugated with an ionizable lipid for designing LNPs for the treatment of diseases of the central nervous system (Ma et al., 2020). To decorate the nanoparticle surface with antibodies, postmodification of preformed LNPs is carried out: in this case, antibodies are most often conjugated with a functionalized PEGylated lipid (Tombacz et al., 2021; Rurik et al., 2022; Zhou et al., 2022; Billingsley et al., 2024).

In the present review, we focus on discussing the progress of improvements in LNP-based systems of delivery of mRNA owing to either alterations of lipid composition or changes in lipid ratios, without touching on the vast realm of protein macromolecules and antibodies. Additionally, we show which functional groups and physicochemical properties of lipids can ensure their targeted delivery to desired organs.

## 2 Biodistribution of LNPs

**FIGURE 1:**
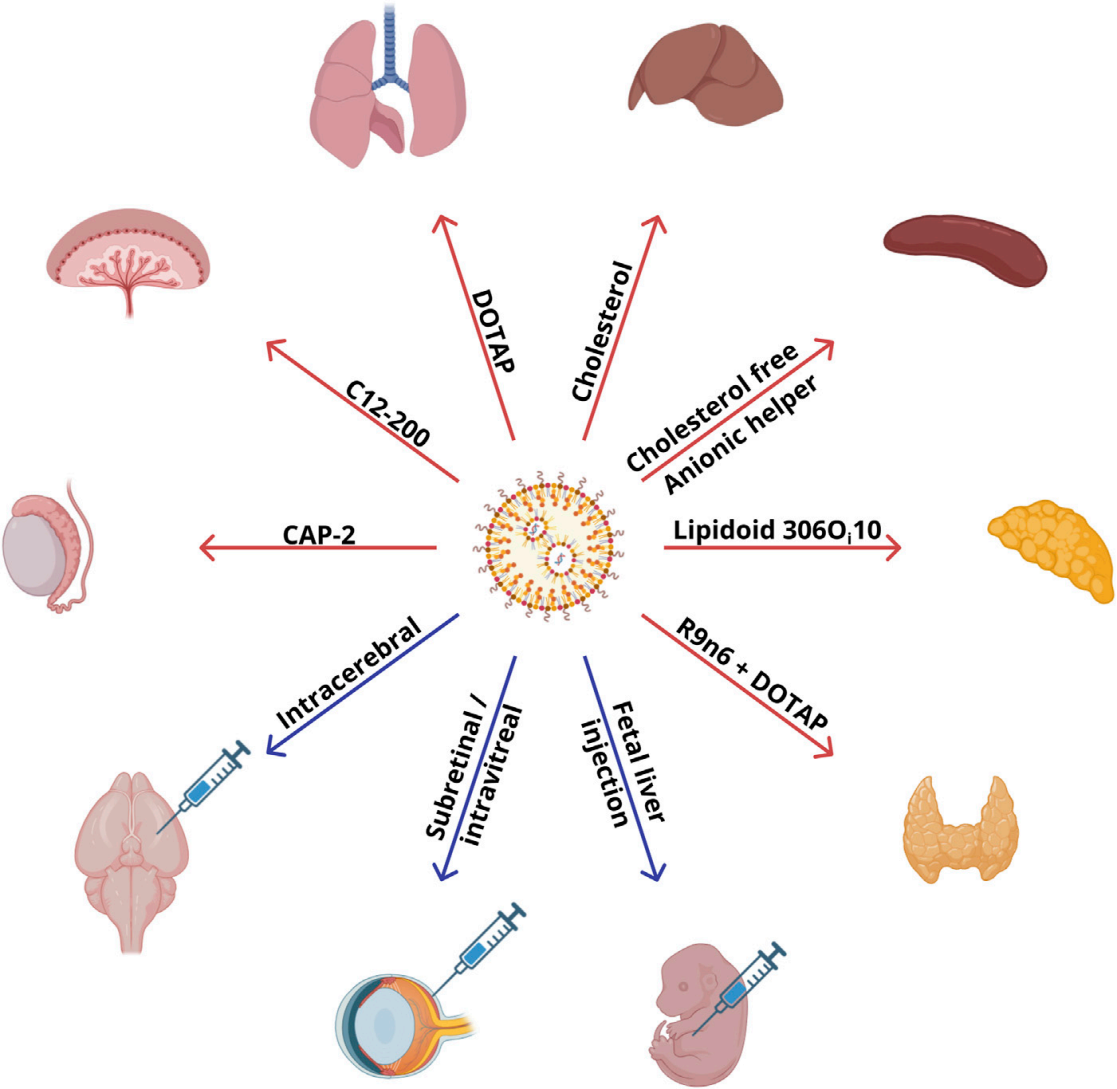
Commercially available lipids and administration routes that are employed in compositions of LNPs ensuring specific targeting to organs.

Biodistribution of particles *in vivo* depends on the route of administration, size, lipid composition of the particles, and the animal model in question. pK_a_ of a particle affects the binding of blood proteins to its surface, thereby also influencing the distribution among organs. Typical biodistribution of LNPs is described in Section 2.1. The dependence of nanoparticle biodistribution on the route of administration and other parameters without changes in lipid composition or helper and ionizable lipids is presented in Sections 2.2, 2.3 (Figure 1).

### 2.1 Typical biodistribution of LNPs

The lion’s share of various LNPs gets distributed to the liver and to a lesser extent to the spleen, and the reason is the presence of a sinusoidal discontinuous endothelium with pores of a ∼100–200 nm diameter in these organs (Kumar et al., 2023). After intravenous administration and entry into the bloodstream, a protein corona forms on the surface of LNPs, and this topic is investigated in detail in Dilliard et al. (2021). The protein corona is capable of influencing the biodistribution of nanoparticles among tissues in various organs owing to the adsorption of the proteins on the tissue surface, leading to internalization via specific receptors. The best-studied mechanism of targeting of LNPs involves their uptake by hepatocytes via binding to low-density lipoprotein (LDL) receptors on their surface (Paunovska et al., 2022). The ApoE protein present in blood plasma is a ligand of LDL receptors and binds to cholesterol on the nanoparticles’ surface and ensures their predominant accumulation in liver cells (Paunovska et al., 2022; Da Silva Sanchez et al., 2022). This mechanism is confirmed by experimental data where LNPs incubated with ApoE have been found to be targeted to hepatocytes (Johnson et al., 2022); on the other hand, particles with a protein corona composed of albumin are distributed preferentially into Kupffer cells instead of hepatocytes (Johnson et al., 2022). Overall, the delivery of LNPs into cells by an ApoE-independent mechanism leads to their accumulation in cells other than hepatocytes (Wadhera et al., 2016). A decrease in the proportion of cholesterol also impairs the delivery of LNPs to hepatocytes (Kawaguchi et al., 2023). Because of the fairly widespread expression of LDL receptor in human tissues (https://www.proteinatlas.org/ENSG00000130164-LDLR/tissue), delivery of nanoparticles containing cholesterol and ApoE (either contained in the particle or immobilized from blood plasma) to all the above tissue types may be promising, e.g., to tissues of the lungs, stomach, liver, and kidneys. The second pathway of LNP uptake in the liver involves Kupffer cell scavenger receptors. For macrophages, there is *in vitro* evidence that they employ clathrin-mediated endocytosis to internalize LNPs (Hou et al., 2020a).

### 2.2 Correlation between the size of LNPs and their biodistribution

The size of LNPs also influences targeting efficiency of delivery. When administered intramuscularly, the nanoparticles end up in the liver and spleen, especially if the LNP size is less than 100 nm (Di et al., 2022). Larger nanoparticles, 320 nm, remain at the injection site because they do not seem to be able to penetrate capillaries effectively (Di et al., 2022). Furthermore, the biodistribution of LNPs may differ among animal species (Lam et al., 2023). It is believed that the optimal particle size for nonhuman primates (50–60 nm) is smaller than that for rodents (70–80 nm), possibly indicating a need for further optimization of LNP size for use in humans (Lam et al., 2023; Hassett et al., 2021).

### 2.3 Uncommon methods of LNP injection

Recent research (Labonia et al., 2023; Hou et al., 2023; Yu et al., 2023) shows that for some tissues/organs, such as the heart, kidneys, and cartilage, a desired protein’s expression can be achieved via injection of an mRNA–LNP complex directly into a target organ (Figure 1). To deliver an mRNA–LNP complex to the retinal pigment epithelium, the subretinal and intravitreal routes of administration have been used, which ensure high expression of a reporter protein (Patel et al., 2019). Expression of the luciferase reporter gene in the retinal pigment epithelium has been observed with the standard four-component composition of LNPs: ionizable lipid:DSPC:cholesterol:DMG-PEG2000 at 50:10:38.5:1.5. The lipid compositions guaranteeing reporter gene expression in the pigment epithelium have contained an ionizable lipid with a tertiary amine (Dlin-MC3-DMA [MC3], Dlin-KC2-DMA [KC2], or 1,2-dioleyloxy-3-dimethylaminopropane [DODMA]). By contrast, nanoparticles based on cationic lipids, N-(4-carboxybenzyl)-N,N-dimethyl-2,3-bis(oleoyloxy)propan-1-aminium (DOBAQ), 1,2-di-O-octadecenyl-3-trimethylammonium propane (DOTMA), 2-dioleoyl-sn-glycero-3-ethylphosphocholine (18:1 EPC), or 1,2-dioleoyl-3-trimethylammonium-propane (DOTAP), have not led to the expression of a reporter protein in the retinal pigment epithelium (Patel et al., 2019). MC3 and KC2 molecules with pK_a_ = 6.4 and two double bonds in each hydrocarbon tail have shown higher transfection activity as compared to DODMA with pK_a_ of 7.0 and one double bond in each lipid tail. Researchers attribute this outcome to the fact that the high unsaturation of hydrocarbon radicals in combination with pK_a_ within the pH range of endosomes (5.5–6.5) promotes membrane destabilization and facilitates the exit of nucleic acids from endosomes (Patel et al., 2019). Most of the reporter protein expression in that study was found in the retinal pigment epithelium and only weak expression in Müller glial cells. Because various types of blindness are associated with the retinal pigment epithelium dystrophy caused by monogenic diseases (Chawla and Vohra, 2023), genetic modification of the retinal pigment epithelium may hold promise for the fight against such diseases. In another study (Ryals et al., 2020), investigators developed nanoparticles based on ionizable MC3 that were able to transfect Müller glial cells, the optic nerve head, and trabecular meshwork with less traumatic (intravitreal) injection. For intravitreal administration, a higher concentration of mRNA is required relative to subretinal administration: 1,500 ng of RNA per dose of the therapeutic agent versus 200 ng. The best efficiency was manifested by particles with 0.5% of PEG and a size of ∼150 nm, which continued to express the fluorescent reporter in retinal cells even on the seventh day (Patel et al., 2019). Particles with a size of 150 nm were too large for phagocytosis, but with a size of <500 nm, they can still be taken up via endocytosis. Of note, reducing particle size to 50–60 nm for retinal delivery impaired reporter protein expression as compared to the larger particles. Similar deterioration of reporter protein expression was also observed when the molar percentage of PEG in the LNPs was increased to 5% (Patel et al., 2019). Five-component particles based on another ionizable lipid, LP01 (Gautam et al., 2023), have been successfully used for gene editing in the retina.

A separate difficult task is the delivery of mRNA therapeutics to the brain or fetal tissue because they are protected by the blood–brain barrier and blood–placental barrier. The passage of LNPs—without additional modifications—through the blood–brain barrier is still a matter of debate (Pardridge, 2023; Pateev et al., 2023). These data suggest that the delivery of LNPs to the brain remains an unsolved problem, and only a few authors have detected their trace amounts (Pateev et al., 2023). After traumatic brain injury, the permeability of the blood–brain barrier is disturbed, and delivery of LNPs up to 100 nm in size to the brain becomes possible (Waggoner et al., 2023). MC3-based four-component nanoparticles carrying luciferase mRNA were administered intravenously in the work just cited, with major distribution to the liver and spleen. When the content of DSPE-PEG was diminished from 1% to 0.1% (and accordingly the particle size was increased), the difference between the damaged and undamaged brain in distribution increased by more than an order of magnitude in the period 4–24 h. After administration of a Cy5-labeled mRNA–LNP complex into the brain via convection-enhanced delivery, the particles diffused only 1 mm away from the injection site and were found in neurons, microglia, and astrocytes (Waggoner et al., 2023). A relatively wide distribution of LNPs in the brain has been achieved only with the help of stereotactic injections of a reporter mRNA–LNP complex (Tuma et al., 2023). Administration of Cre mRNA as an mRNA–LNP complex into the striatum and hippocampus of Ai14 mice in that report led to the expression of the tdTomato reporter within a month in these brain regions (Tuma et al., 2023). Expression of the reporter protein was observed within a radius of up to 3 mm from the injection site, with the greatest intensity in the range 750–1,250 μm in the striatum, and as far away as 2,500 μm (with the greatest intensity in the range 1,250–2,250 μm) in the hippocampus; in this context, the system of single guide RNA and Cas9 induced tdTomato expression in fewer cells as compared to the Cre mRNA system. Furthermore, the expression of the reporter protein was observed there in different types of cells: neurons, astrocytes, and microglia.

LNPs also do not cross the blood–placental barrier (Pateev et al., 2023). To deliver mRNA within LNPs into embryonic tissues, direct injection into the liver of embryos is performed in pregnant mice (Gao et al., 2023). This route of administration leads to wide biodistribution of LNP-bound mRNA throughout organs of the embryo (Cy5 fluorescence of mRNA is observed in the heart, lungs, liver, kidneys, gastrointestinal tract, and brain) and is also detectable in the liver of a pregnant mouse but not other organs. After administration of Cre mRNA *in utero* to Ai14 mice in that study, mTomato fluorescence was observed in the same organs, with the exception of the brain, indicating the entry of the LNPs into the brain without the expression of the reporter protein (Gao et al., 2023). Notably, in that paper, of all embryonic organs, the highest percentage of cells expressing the reporter protein was observed in the heart: up to 7% (Gao et al., 2023). An analysis of embryonic cell populations after the administration of LNPs there revealed that CD90-positive stem cells get transfected most often in all organs (Gao et al., 2023).

Switching to the intratracheal method of LNP delivery (Massaro et al., 2023) has made it possible to eliminate the expression of the reporter protein in the spleen and liver and to ensure the delivery of LNPs that are based on ionizable lipid MC3 and on phospholipid DPPC (1,2-dipalmitoyl-*sn*-glycero-3-phosphocholine) exclusively to the lungs. An analysis of lung cell populations there indicated that Cy5-labeled mRNA is detectable in approximately half of all type 1 and type 2 alveolar epithelial cells as well as in half of fibroblasts. Of note, targeting to the lungs after intratracheal administration was not dependent on particle size. In Massaro et al. (2023), nanoparticle size was 34 nm; in another study (Zhang et al., 2020), where the particles were also effectively targeted, their size was ∼150 nm. Thus, several different compositions of four-component particles based on the MC3 lipid and helper lipid DOPE or DPPC have turned out to be targetable to lungs when administered intratracheally (Zhang et al., 2020). It should be pointed out that in both studies on delivery to lungs (Massaro et al., 2023; Zhang et al., 2020), the proportion of a helper lipid (15–20 mol%) was higher as compared to standard (10 mol%) systems of LNPs, possibly indicating the necessity of increasing the helper lipid content for delivery to lungs.

The route of administration also has a significant impact on kinetics of nanoparticle distribution. In Pardi et al. (2015), effects of various administration routes were studied toward the pharmacokinetics of four-component LNPs based on new ionizable lipid L319. After subcutaneous or intradermal administration of mRNA with LNPs, reporter protein expression was observed at the injection site, whereas after intramuscular, intraperitoneal, or intravenous (retro-orbital) administration, at the injection site and in the liver. Upon intratracheal administration, the expression was noted in the lungs and liver. In that paper, intradermal administration caused the most prolonged reporter protein expression: 9 days. In all likelihood, this outcome is due to longer retention of LNPs in the dermis. There are reports of other patterns for nanoparticle distribution. For instance, in Huysmans et al. (2019), it was demonstrated that when administered subcutaneously, LNPs remain at the injection site, with partial migration to a draining lymph node after 24 h. Moreover, additional use of electroporation immediately after the subcutaneous administration of LNPs enhanced the expression of the reporter protein by two orders of magnitude (Huysmans et al., 2019).

Therefore, the route of administration of RNA–LNP therapeutics has a significant impact on their biodistribution. Existing research articles show that even without special manipulation of lipid composition, intracerebral, subretinal/intravitreal, or intratracheal administration of LNPs causes specific delivery to the brain, retina, or lungs, respectively.

## 3 LNP targeting based on commercially available lipids

**FIGURE 2:**
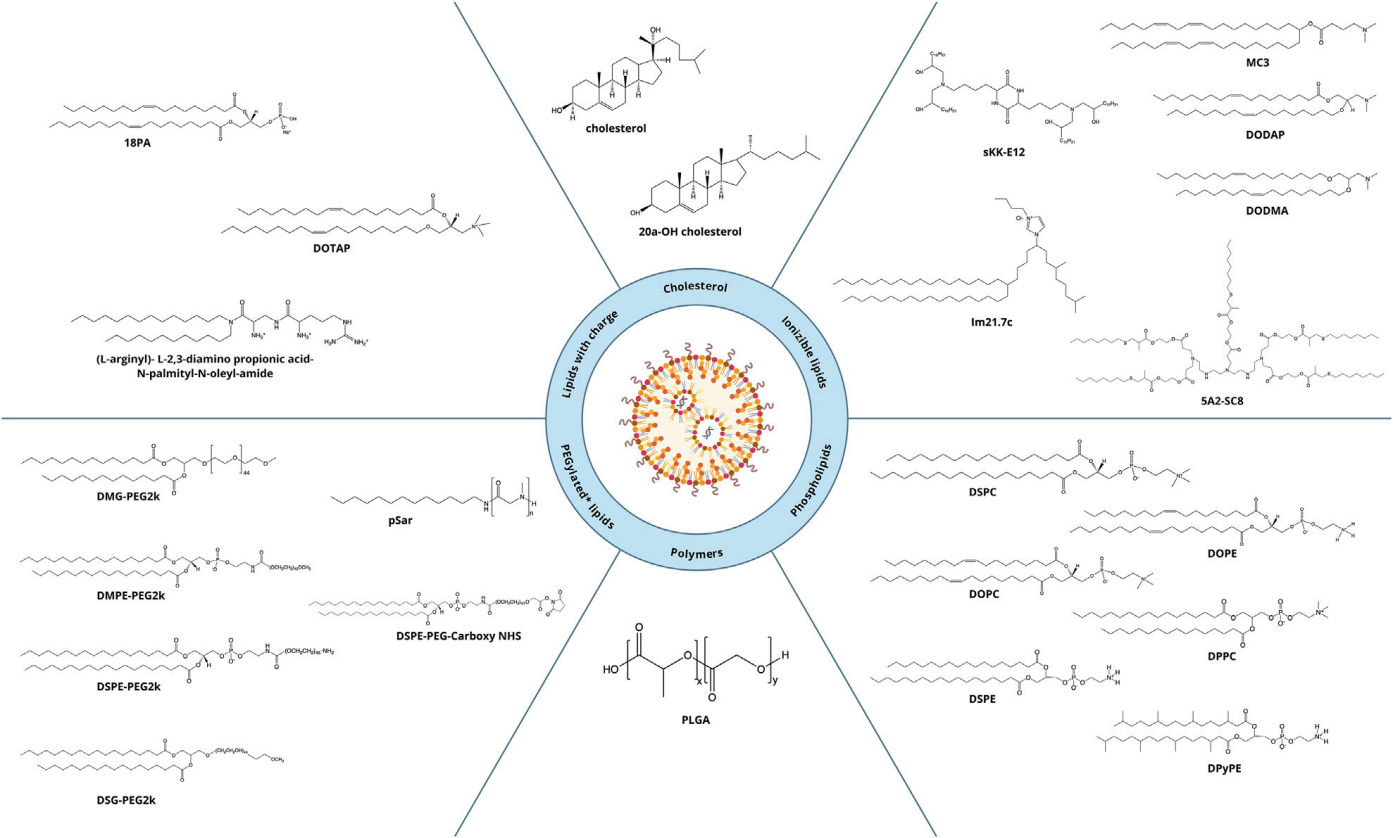
Lipids ensuring targeting of LNPs to desired organs.

An important aspect of this review is alteration of LNP targeting by changes in lipid composition by the most common methods of administration: intravenous and intramuscular. To alter the targeting of nanoparticles, researchers change components (the helper phospholipid, cholesterol, and/or PEGylated lipid) from classic lipids either to commercially available similar lipids (discussed in this section) or to specially synthesized lipids (to be addressed in Section 4). We identified commercially available lipids that constitute formulations targeting specific organs (Figure 2) and examined in detail i) the distribution across organs and cell populations, ii) routes of administration and doses, and iii) chemical and physical properties of successful compositions (Table 1).

**TABLE 1:** Specific targets of LNPs based on commercially available lipids.

Molar proportion of lipids used, %	Administration	Distribution or expression of mRNA among targeted and other organs	Distribution by cell population	Physicochemical properties of particles	Detection	Reference
50 MC3 10 DSPC 38.5 Сholesterol 1.5 DMG-PEG2000	Subretinal FLuc mRNA 200 ng per mouse	Eyes ∼ 100% Not measured in other organs	Retinal pigment epithelium Muller glia	Size 148.6 ± 68.4 nm PDI 0.173 EE^a^ 97.9%	IVIS 24 h IHC 24 h	Patel et al. (2019)
50 MC3 10-11 DSPC 38.5–39.5 Cholesterol 0.5 DMG-PEG2000	Subretinal FLuc/mTomato mRNA 200 ng per mouse Intravitreal FLuc/mTomato mRNA 1.5 mg per mouse	Eyes ∼ 100% Not measured in other organs	Retinal pigment epithelium Ganglion cell layer Ciliary body Trabecular meshwork Optic nerve head	EE 96.2% (for 38.5% cholesterol) EE 97.8% (for 39.5% cholesterol)	IVIS 24 h Fundus photography 7 day IHC day 7	Ryals et al. (2020)
36.8 MC3 23.8 DOPE 38.2 Cholesterol 1.2 DMG-PEG2000	Intracerebral injections cre mRNA 0.375 mg per mouse	Brain Striatum Hippocampus	Neurons Astrocytes Microglia	Size 162.4 nm PDI 0.219	IHC day 21	Tuma et al. (2023)
50 MC3 10 DSPC 38.5 Cholesterol 1.5 DSPE-PEG2000	Intravenous FLuc mRNA 0.5 mg/kg	Injured brain 6% Liver 86%, Spleen 6% Heart <1% Lungs <2%	Neurons Astrocytes Microglia	Size 50 nm EE 97.0%	Luminescence 24 h	Waggoner et al. (2023)
35-45.4 MC3 11-23.8 DOPE 37.7-49.5 Cholesterol 1.2-2.5 DMG-PEG2000	Fetal liver injection cre mRNA 6.25 ng per fetus	Fetus liver ∼20%–25% Fetus spleen ∼20% Fetus lungs ∼ 15%–20% Fetus kidneys ∼20%–30% Fetus heart ∼ 20% Fetus gut ∼ 20%	Endothelial cells Leukocytes Neurons, Glia, Skeletal muscle Stem cells Proliferating cells Erythroid cells Epithelial cells	Size 112–140 nm PDI ∼ 0.1 EE 76.3% (for 37.7% cholesterol) EE 76.9% (for 38.2% cholesterol) EE 74.7% (for 46.5% cholesterol) EE 82.9% (for 49.5% cholesterol)	IVIS 48 h FACS 48 h	Gao et al. (2023)
	Fetal liver injection Cy7-labeled mRNA 625 ng per fetus	Fetus liver ∼20% Fetus lungs ∼18% Fetus kidneys ∼15% Fetus heart ∼16% Fetus gut ∼18% Fetus brain ∼9%			IVIS 3, 6, 24 h	
42.6 MC3 17 DPPC 39 Cholesterol 2.5 DSPE-PEG2000	Intratracheal Cy5-labeled FLuc mRNA 1.5 mg/kg	Lungs 100%	Alveolar epithelial cells 1 55% Alveolar epithelial cells 2 65%, Fibroblasts 47.4%	Size 34.2 ± 0.8 Zeta 11.5 ± 2.4 PDI 0.29 ± 0.08 EE 90.0%	IVIS 2, 24, 48 h FACS 24 h	Massaro et al. (2023)
40 MC3 16-20 DOPE 39-43 Cholesterol 1 DMPE-PEG2000	Intratracheal FLuc mRNA 1.5 µg per mouse	Lungs ∼100% Liver n.d.^b^ Heart n.d. Kidneys n.d.		Size 60 nm Zeta −7 mV pK_a_ 6	IVIS 6 h	Zhang et al. (2020)
60 MC3 20 DPPC 19 Cholesterol 1 DMG-PEG2000 or 1 DMPE-PEG2000	Intratracheal FLuc mRNA 1.5 µg per mouse	Lungs ∼100% Liver n.d. Heart n.d. Kidneys n.d.		Size 50 nm Zeta −5 mV pK_a_ 5.7 EE 92.0%	IVIS 6 h	Zhang et al. (2020)
35 306O_10_ 40 Ceramide 22.5 Cholesterol 2.5 DMG-PEG2000	Intravenous FLuc mRNA 0.25 mg/kg	Liver 80% Spleen 15% Lungs 5%		Size 120 nm Zeta −5 mV	IVIS 3 h	LoPresti et al. (2022)
35 306O_10_ 40 PA 22.5 Cholesterol 2.5 DMG-PEG2000	Intravenous FLuc mRNA 0.25 mg/kg	Spleen 75% Liver 5% Lungs 5%		Size 120 nm Zeta −7.5 mV	IVIS 3 h	LoPresti et al. (2022)
35 306O_10_ 40 DOTAP 22.5 Cholesterol 2.5 DMG-PEG2000	Intravenous FLuc mRNA 0.25 mg/kg	Lung 80% Liver 1% Spleen 15%		Size 130 nm Zeta −4.5 mV	IVIS 3 h	LoPresti et al. (2022)
35 cKK-E12 44.5 DDAB 18 Cholesterol 2.5 DMG-PEG2000	Intravenous Cre mRNA 1.3 mg/kg	Lungs	Lung endothelial cells Lung dendritic cells Lung T cells Lung B cells Monocytes	Size 106 nm Zeta 1.81 mV pK_a_ 7.7 EE 94.7%	FACS 96 h	Radmand et al. (2023)
30 cKK-E12 39 DOTAP 30 Cholesterol 1 DMG-PEG2000	Intravenous Cre mRNA 0.3 mg/kg	Lungs 25% Liver 60% Pancreas 10%		Size 80 nm Zeta 15 mV	IVIS 48 h	Kauffman et al. (2018)
38.5 4A3-SC8 30 BMP 30 Cholesterol 1.5 DMG-PEG2000	Intravenous FLuc mRNA 0.03 mg/kg	Spleen 80% Liver 20%		Size 130 nm Zeta −4 mV EE 92.0%	IVIS 6 h	Alvarez-Benedicto et al. (2022)
47.6 MC3 9.5 DSPC 4.8 DOPS 37 Cholesterol 1.4 DMG-PEG2000	Intravenous FLuc mRNA 0.05 mg/kg	Spleen + SCLNs^c^ 90% Liver 10%		Size 95 nm Zeta −2.58 mV pK_a_ 6.31	IVIS 6 h	Luozhong et al. (2022)
52.5 C18-PS7.5 DOPS/PS 40 Cholesterol	Intravenous FLuc mRNA 1 μg	Spleen		Size 107 nm Zeta −12.4 mV EE 86.9%	IVIS 6 h	Gomi et al. (2023)
50 MC3 10 DSPG 38.5 Cholesterol 1.5 DMG-PEG2000	Intravenous mCherry mRNA 0.25 mg/kg	Spleen Bone marrow	Hepatocytes liver sinusoidal endothelial cells Kupffer cells	Size 90 nm Zeta −19 mV EE 93.0%	FACS 24 h	Pattipeiluhu et al. (2022)
19 A2-SC8 19 DOPE 38 Cholesterol 4 DMG-PEG2000 40 DODAP	Intravenous Cy5-labeled mRNA 0.5 mg/kg	Liver 60% Spleen 20% Lungs 20% Heart n.d. Kidneys n.d.		Size 154.6 nm Zeta −3.82 mV PDI 0.115	IVIS 6 h	Dilliard et al. (2021)
	Intravenous FLuc mRNA 0.1 mg/kg	Liver ∼100% Spleen 20% Lung n.d. Heart n.d. Kidneys n.d.			IVIS 6 h	
16.7 5A2-SC8 16.7 DOPE 33.3 Cholesterol 3.3 DMG-PEG2000 30 18PA	Intravenous Cy5-labeled mRNA 0.5 mg/kg	Spleen 45% Liver 55% Lungs n.d. Heart n.d. Kidneys n.d.		Size 167.8 nm Zeta −1.84 mV PDI 0.144	IVIS 6 h	Dilliard et al. (2021)
	Intravenous FLuc mRNA 0.1 mg/kg	Spleen ∼100% Liver n.d. Lungs n.d. Heart n.d. Kidneys n.d.			IVIS 6 h	
11.9 5A2-SC8 11.9 DOPE 23.8 Cholesterol 2.4 DMG-PEG2000 50 DOTAP	Intravenous Cy5-labeled mRNA 0.5 mg/kg	Lungs 75% Liver 20% Spleen 5% Heart n.d. Kidneys n.d.		Size 114.8 nm Zeta −0.89 mV PDI 0.159	IVIS 6 h	Dilliard et al. (2021)
	Intravenous FLuc mRNA 0.1 mg/kg	Lungs ∼100% Liver n.d. Spleen <1% Heart n.d. Kidneys n.d.			IVIS 6 h	
n.s.^d^ MC3 n.s. DSPE n.s. DMG-PEG2000 n.s. DOTAP n.s. PLGA (DOTAP:MC3 = 3:1)	Intravenous Cy5-labeled mRNA 6.6 µg per mouse	Lungs 20% Liver ∼70% Spleen 10% Kidneys <3% Heart n.d.		Size ∼180 nm PDI ∼0.2	IVIS 4 h	Meyer et al. (2022)
	Intravenous FLuc mRNA 6.6 µg per mouse	Lungs ∼80% Spleen ∼15% Heart <5% Kidneys <5% Liver <5%				
n.s. MC3 n.s. DSPE n.s. DMG-PEG2000 n.s. DOTAP n.s. PLGA (DOTAP: MC3 = 1:1)	Intravenous Cy5-labeled mRNA 6.6 µg per mouse	Spleen 20% Liver ∼70% Lungs ∼5% Kidneys <10% Heart n.d.		Size ∼170 nm PDI 0.15	IVIS 4 h	Meyer et al. (2022)
	Intravenous FLuc mRNA 6.6 µg per mouse	Spleen 50% Lungs ∼40% Liver <10% Kidneys <5% Heart <5%				
40 IM21.7c 30 DODMA 10 DPyPE 18.5 Cholesterol 1.5 DSG-PEG2000	Intravenous FLuc mRNA 7.5 µg per mouse	Lungs >95% Spleen ∼ 3% Liver <1%		Size 97 ± 1 nm PDI 0.089 Zeta 12 mV EE 98.0%	Luminescence 24 h	Gueguen et al. (2024)
50 MC3 10 DSPC 38.5 Cholesterol 1.2 DMG-PEG2000 0.3 DSPE-PEG2000-Carboxy NHS	Subretinal Cre mRNA 500 ng per mouse		Retinal pigment epithelium Outer nuclear layer Photoreceptor nuclei	Size 60 nm PDI 0.1 EE 97.0%	IHC 48 h	Gautam et al. (2023)
50 MC3 8.5 DOPC 40 Cholesterol 1.5 DMG-PEG2000	Intramuscular FLuc mRNA 2 μg per mouse	Liver ∼ 35 Muscle ∼65%		Size 100 nm Zeta −3.85 ± 1.59 mV PDI ∼ 0.1 EE 97.0%	Luminescence of homogenized organs 4.5 h	Kawaguchi et al. (2023)
	Subcutaneous FLuc mRNA 2 μg per mouse	Liver ∼ 1% Skin ∼ 99%				
50 Cationic lipid^e^ 49 DPyPE 1 DSPE-PEG2000	Intravenous FLuc mRNA 1.5 mg/kg	Lung	Alveolar capillary cells Monocytes Neutrophils	Size 87.61 nm PDI 0.082 Zeta 8 mV EE >95.0%	Cenomics: single-cell RNA sequencing 6 h	Radloff et al. (2023)
60 DOPE 28.5 DODAP 10 Cholesterol 1.5 DMG-PEG2000	Intravenous FLuc mRNA 0.8 µg/kg	Spleen >95% Liver <3% Lungs <1%	Macrophages ∼50% Dendritic cells ∼40% B cells ∼5% T cells n.d.	Size 150 nm Zeta −10 mV	Luminescence 24 h	Shimosakai et al. (2022)
50 cKK-E12 35.5 DOPE 2.5 DMPE-PEG2000 12.5 20a-OH Cholesterol	Intravenous Cre mRNA 0.25 µg/kg	Liver Spleen Heart n.d. Kidneys n.d. Pancreas n.d. Lungs n.d. Bone marrow n.d.	Liver Endothelial cells: 90% Hepatocytes: 20% Kupffer cells: 90% Immune cells: 55%	Size 80.1 nm PDI 0.16 pK_a_ 5.6 EE 83.0%	FACS 72 h	Paunovska et al. (2019)
50 cKK-E12 35.5 DOPE 2.5 DMPE-PEG2000 12.5 20a-OH Cholesterol	Intravenous Cre mRNA 0.3 mg/kg	Liver	Endothelial cells Kupffer cells T cells Monocytes B cells Macrophages	Size 88 nm PDI 0.228 pK_a_ 6.8	FACS 72 h	Hatit et al. (2023)
40 DODMA 10 DSPC 45 Cholesterol 5 polysarcosine	Intravenous FLuc mRNA 10 µg per mouse	Spleen ∼ 50% Liver ∼ 50% Heart n.d. Lungs n.d. Kidneys n.d.		Size ∼125 nm PDI 0.25 EE 92.0%	IVIS 6 h	Nogueira et al. (2020)

^a^ EE = encapsulation efficiency

^b^ n.d. = not detected

^c^ SCLNs = superficial cervical lymph nodes

^d^ n.s. = not specified

^e^ (L-arginyl)-L-2,3-diamino propionic acid-N-palmityl-N-oleyl-amide

### 3.1 Helper lipids

The classic composition of LNPs for RNA delivery contains a zwitterionic (neutral) helper phospholipid. In the last few years, many studies were focused on targeted delivery of LNPs to organs (mainly to the spleen and lungs) via changes in the charge of the nanoparticles, which is determined by the helper lipid. This strategy is called Selective ORgan Targeting (SORT), and the lipid responsible for the selective delivery of nanoparticles to organs is called a SORT-lipid (Cheng et al., 2020). Besides, the SORT strategy can be applied in two ways: complete replacement of a helper lipid or partial replacement (introduction of a fifth component).

In LoPresti et al. (2022), it was found that complete replacement of a zwitterionic helper lipid with an anionic one induces greater accumulation of LNPs in the spleen as compared to the liver, whereas replacement with a cationic helper lipid leads to LNP accumulation in lungs. It must be noted that the nature of the helper lipid in that report did not have as much influence on nanoparticle biodistribution as did the charge of the lipid’s polar head.

In Cheng et al. (2020); LoPresti et al. (2022); Radmand et al. (2023), it has been convincingly proven that the use of a cationic helper lipid drives the accumulation of LNPs in lungs. Besides, in Radmand et al. (2023); Kauffman et al. (2018), it has been demonstrated that the greatest accumulation of LNPs occurs in endothelial cells, which are most accessible to the bloodstream. The effect of cationic lipid DOTAP’s proportion in LNPs on the biodistribution of nanoparticles is intriguing, as described in Cheng et al. (2020). For example, LNPs devoid of DOTAP accumulated in the liver; LNPs having a DOTAP content of 10%–15% predominantly ended up in the spleen, whereas with an increase in the DOTAP content above 50%, the LNPs were detectable almost exclusively in lungs. A similar change of the organ showing the highest accumulation (liver, spleen, or lungs) when the proportion of a cationic lipid was increased was documented for other cationic helper lipids: DDAB (dimethyl-dioctadecyl-ammonium bromide) and EPC.

The use of an anionic helper lipid leads to the accumulation of LNPs in the spleen after intravenous administration (Alvarez-Benedicto et al., 2022; Luozhong et al., 2022; Gomi et al., 2023). In Luozhong et al. (2022), it was found that when 1,2-dioleoyl-*sn*-glycero-3-phospho-L-serine (DOPS) served as the anionic lipid, aside from the accumulation of LNPs in the spleen, their specific accumulation was registered in superficial cervical lymph nodes, in contrast to LNPs containing phosphatidic acid. Authors of Gomi et al. (2023). demonstrated that phosphatidylserine-containing LNPs are mainly taken up by hepatic phagocytes. In this context, the presence of an ionizable lipid in the LNPs enhanced the uptake of the nanoparticles by cells. It is worth mentioning that specific delivery of phosphatidylserine-containing LNPs to lymphoid organs was achieved there only with intravenous administration.

As stated above, targeted delivery of LNPs to organs is determined by the charge, not by the nature of the polar group in a helper lipid. For instance, for nanoparticles containing anionic phosphatidylserine, phosphatidylglycerol, or phosphatidic acid, researchers have observed more specific delivery to the spleen (60%, 50%, and 75%, respectively) than to the liver (20%, 45%, and 5%, respectively) (LoPresti et al., 2022). On the other hand, another group of authors (Pattipeiluhu et al., 2022) has reported that for LNPs containing phosphatidylglycerol, accumulation in the liver occurs predominantly in sinusoidal endothelial cells and Kupffer cells, in contrast to phosphatidylcholine-containing LNPs, whose accumulation takes place equally in hepatocytes, liver sinusoidal endothelial cells, and Kupffer cells.

The introduction of an additional charged helper lipid as a fifth component also enables investigators to alter targeting efficiency of LNPs. Not so long ago (Dilliard et al., 2021), scientists achieved almost 100% reporter mRNA expression in the liver, spleen, or lungs by means of an additional neutral, negatively, or positively charged lipid, respectively. Targeting to an organ depended there on apparent pK_a_ of nanoparticles: at pK_a_ < 5, the particles accumulated mainly in the spleen; at pK_a_ of 6.2–6.4, mainly in the liver; whereas at pK_a_ > 11, mainly in lungs. It is noteworthy that at pK_a_ of 6.5–11, there was selective distribution of LNPs into the lungs and spleen at different levels, indicating a nonobvious dependence of the LNP biodistribution on the charge. It is likely that pK_a_ can serve as a good predictor of bioactivity. For classic four-component LNPs, apparent pK_a_ was 6.3–6.6 in the above study, depending on the ionizable cationic lipid used. The addition of an anionic lipid reduced pK_a_, whereas the addition of a cationic lipid increased pK_a_, which affected the interaction with plasma proteins and the formation of the protein corona (Dilliard et al., 2021). Results of their proteomic analysis of the protein corona suggested that targeting to the liver takes place with predominance of the ApoE protein in the protein corona; to lungs, with predominance of the β2-GPI protein; and to the spleen, with predominance of the VTN protein. Incubation of these proteins with the LNPs *in vitro* also enabled specific LNP targeting (Dilliard et al., 2021).

Those authors proposed a three-stage mechanism underlying the targeting of a five-component LNP (Dilliard et al., 2021). The first stage is the dissociation of the PEGylated lipid from the surface of LNPs; the second is the binding of SORT-lipid to blood proteins; and the third stage is the binding of proteins (adhering to the particle) to tissue-specific receptors. In the case of PEG with a longer aliphatic anchor, which shows worse desorption, the expression of the reporter protein in all organs was noticeably lower, thus confirming the above mechanism. In another study (Meyer et al., 2022), LNPs containing a positively charged group of the additional lipid and a PLGA polymer also got distributed into lungs and the spleen, and the distribution was influenced by the ratio of a cationic lipid (DOTAP) to an ionizable lipid (MC3). Of note, switching the method of mRNA introduction from encapsulation to adsorption made it possible to achieve up to 80% expression of a target protein in the lungs when composition with a DOTAP/MC3 ratio of 1:1 was employed, and up to 50% expression in the spleen with composition featuring a DOTAP/MC3 ratio of 3:1. Nanoparticles carrying adsorbed mRNA had a size of ∼200 nm and did not accumulate in kidneys during excretion, likely indicating the suitability of such particles for subsequent pharmaceutical research and development. In addition, these nanoparticles did not contain cholesterol, and Cy5-labeled mRNA was detectable but not expressed in the liver; this finding may be predictive of the behavior of other cholesterol-free particles. These data are consistent with another study (Gueguen et al., 2024), where LNPs of five-component composition containing cationic lipid IM21.7c also got distributed into lungs and the spleen (and very little into the liver), and >95% of the reporter protein was expressed in the lungs when the LNPs were administered intravenously. The zeta-potential of the LNPs containing cationic lipid IM21.7c was positive, in contrast to the previously obtained nanoparticles (Dilliard et al., 2021), which also got distributed into lungs and the spleen but had negative zeta-potential. We believe that zeta-potential has insufficient predictive value to determine a target organ.

Thus, the addition of a charged lipid into LNPs affects pK_a_ of the particles, which in turn determines the formation of a protein corona on the surface of the LNPs and accordingly their subsequent distribution throughout organs, which is modulated by proteins of the corona. This principle allows to redirect particles between the liver, spleen, and lungs through the addition of neutral, negatively charged, or positively charged lipids, respectively. It is possible that a combination of an administration route with charged lipids can ensure targeting to new types of tissues and cells.

### 3.2 Cholesterol

The LNPs utilized in most studies contain a substantial amount of cholesterol (up to 40 mol%). In Kawaguchi et al. (2023), researchers investigated the effect of the cholesterol content on physicochemical properties of LNPs and on the magnitude of expression and distribution of a reporter protein with various routes of administration. In that study, LNPs were obtained with the classic cholesterol content of 40%, as were LNPs with a lowered cholesterol content of 20% and 10%. From the standpoint of physicochemical properties, the nanoparticles of all types turned out to be almost identical. Their sizes ranged from 75 to 140 nm, with a relatively narrow size distribution (polydispersity index [PDI] <0.25). It is worth noting that LNPs of the classic composition were smaller compared to the others and had a narrower size distribution. At the same time, the stability of the obtained LNPs decreased with the diminishing proportion of cholesterol: within a week, particles with 10% of cholesterol increased in size while losing encapsulated mRNA. Cholesterol only weakly affects the degree of mRNA encapsulation into nanoparticles. Usually, the correlation between the cholesterol amount and mRNA loading is within systematic error, and therefore it cannot be said definitively that cholesterol increases the loading. A weak correlation between RNA encapsulation and the level of cholesterol in LNPs has been observed only in two studies (Kawaguchi et al., 2023; Zhang et al., 2020). In Kawaguchi et al. (2023), the effect of cholesterol was demonstrated the most clearly because concentrations of the other components, except for phospholipid, were fixed. Taking into account their reported experimental errors, encapsulation efficiency remained constant at the ratios used. The only parameter that cholesterol affected is the stability of the resulting nanoparticles. In the same work (Kawaguchi et al., 2023), it was noted that with a decrease in the cholesterol amount, the nanoparticles tend to increase in size over time, and they also have a higher PDI and their encapsulation efficiency declines over time.

For subcutaneous and intramuscular administration of the LNPs, a decrease in the cholesterol content reduced the biodistribution of the nanoparticles to the liver and the expression of the reporter protein there, whereas at the injection site, the level of reporter protein expression did not change. This evidence suggests that the creation of LNPs that do not end up in the liver requires reducing the amount of (or replacing) cholesterol, in agreement with the results from Radloff et al. (2023), which indicates that the use of cholesterol-free three-component LNPs when administered intravenously leads to reporter protein expression almost exclusively in lungs, with small levels in the spleen. Reducing the cholesterol content also improved the targeting of LNPs to the spleen when the LNPs were based on known lipids and were administered intravenously (Radloff et al., 2023). In the optimized composition of LNPs in Shimosakai et al. (2022), which showed >95% efficiency of LNP delivery to the spleen, the cholesterol content was reduced to 10 mol%, whereas the concentration of a helper lipid was increased to 60 mol%. Among splenocytes, the LNPs got successfully distributed into antigen-presenting cells but not T lymphocytes (Shimosakai et al., 2022).

Modified cholesterol can also be employed for LNP targeting. In Paunovska et al. (2019), scientists assessed the effect of cholesterol modifications on the delivery of Cre mRNA within LNPs into different types of cells of Ai14 mice in seven organs: the liver, spleen, heart, kidneys, lungs, pancreas, and bone marrow. Those authors used oxidized forms of cholesterol, where rings or the tail of cholesterol were modified with a hydroxyl or ketone group. The distribution of these LNPs among cell and tissue types did not depend on the cholesterol being tested; tdTomato-positive cells were found in the spleen and liver; in other tissues, the percentage of such cells was negligible. The proportion of liver cells expressing tdTomato was 100%, 80%, 60%, and 20% among Kupffer cells, endothelial stem cells, immune cells, and hepatocytes, respectively, whereas in the spleen, 17%, 9%, and 3% among T cells, B cells, and macrophages, respectively (Paunovska et al., 2019). The administration of LNPs containing modified 20α-hydroxycholesterol enhanced reporter protein expression in endothelial cells, hepatocytes, Kupffer cells, and liver immune cells by an order of magnitude as compared to LNPs containing unmodified cholesterol (Paunovska et al., 2019). Authors of Hatit et al. (2023) have obtained additional data on the use of 20α-hydroxycholesterol in LNPs thus showing that the observed effect is not due to a change in physicochemical properties of the LNPs but is explained by biological effects. Their LNPs containing a mixture of stereoisomers, when entering a cell, activated genes associated with phagocytosis and inflammation to a greater extent. Consequently, replacing unmodified cholesterol with stereopure 20α-hydroxycholesterol may be promising for increasing the expression of a target protein.

Thus, cholesterol is necessary for membrane stability but at the same time directs LNPs to the liver. Replacing cholesterol with analogs does not affect the biodistribution but may influence the efficiency of cell transfection. Accordingly, to redirect particles from the liver to other organs, one possible solution is to devise cholesterol-free formulations or utilize additional features that will ensure targeting to a desired organ while promoting stability of the nanoparticles’ membrane.

### 3.3 PEGylated lipids

The introduction of PEG into lipid composition allows for self-assembly of LNPs and reduces nanoparticle aggregation and heterogeneity owing to steric stabilization. Furthermore, PEG improves the stability and durability of LNPs because of the formation of a shell that stabilizes the membrane (Hald et al., 2022). The downside of PEG addition is that an overly stable membrane prevents LNPs from fusing with an endosome, and hence the mRNA is unable to enter the cell. Some articles (Gueguen et al., 2024; Ryals et al., 2020; Lam et al., 2023) suggest that reducing the percentage content of a PEGylated lipid improves the expression of a target protein in animals. By varying the molar percentage and molar mass of PEG, an investigator can control the size and size distribution of the resultant nanoparticles. In one study (Sarode et al., 2022), a series of PEGylated lipids was investigated that differed in length (550–2000 Da) and architecture (linear or branched) of the polymer chain and in the nature of the lipid moiety (phosphoglyceride, diglyceride, or ceramide). Those authors revealed that LNPs containing a PEGylated lipid based on phosphoglycerides are characterized by a pronounced dependence of particle size on the molar proportion and molecular weight of PEG in contrast to lipids based on diglycerides and ceramides. Among particles with a high content of a long-chain PEGylated lipid, the size distribution was broad, which those authors attributed to the formation of a subpopulation of PEGylated lipid micelles. There was no obvious impact of the lipid moiety on the size of the LNPs.

Of note, the use of LNP therapeutics containing a PEGylated lipid can cause unwanted immune responses in the body (Tenchov et al., 2021; Shiraishi and Yokoyama, 2019). In particular, the emergence of accelerated blood clearance has been documented. In the blood, anti-PEG antibodies appear that quickly remove the administered PEGylated nanoparticles. As a result, the effectiveness of the mRNA therapeutic agent diminishes, and there is a need for repeated administration of the therapeutic. Thus, the incorporation of a PEGylated lipid into LNPs can be an obstacle to repeated use of mRNA–LNP therapeutics. As an alternative to PEG, a polysarcosine (pSar) has been investigated, which is a polypeptide derived from sarcosine and possesses all the properties of PEG (Birke et al., 2018). Moreover, a polysarcosine does not induce an immune response even after repeated administration, in contrast to PEG.

Polysarcosines can serve as analogs of PEG (Nogueira et al., 2020). Just as for PEG, an increase in the molar proportion of a polysarcosine within LNPs reduces particle size: this pattern is observed when the molar proportion of a polysarcosine within LNPs changes from 1.5% to 10%, and the shorter the polysarcosine, the more pronounced is the effect. In the article just cited, the use of the shortest polysarcosine called pSar11, containing 11 units, within LNPs diminished particle size from 300 to 125 nm, whereas the addition of the longest polysarcosine (pSar65, containing 65 units) into the LNPs led to a decrease in particle size from 150 to 75 nm. With increasing length of the polysarcosine component of the LNPs, the accumulation of the particles shifted from the spleen to the liver. Those authors ascribe this redistribution of LNPs to their size: larger particles with a shorter polysarcosine accumulate more efficiently in the spleen. A similar redistribution pattern was registered with an increase in the proportion of a polysarcosine of the same chain length within the LNPs: an increase in the proportion of the polysarcosine reduced particle size and hence shifted their accumulation from the spleen to the liver. Additionally, LNPs based on a polysarcosine, as compared to conventional PEG, have lower reactogenicity, as shown in experiments on mice (Nogueira et al., 2020). A recent study (Gautam et al., 2023) addressed various modifications of PEG within LNPs. For subretinal delivery, LNPs were tested containing additional PEG modified with a carboxyl group, an amine group, or a carboxyester group. Depending on the charge, the LNPs showed different tropism for eye photoreceptor cells. Control four-component LNPs induced tdTomato expression only in the retinal pigment epithelium, whereas LNPs with all types of modified PEG were able to transfect nuclei of cone and rod photoreceptor cells (Gautam et al., 2023). In these experiments, LNPs containing PEG with the carboxyester group caused expression of the reporter protein in the outer segment of a photoreceptor, in the nucleus, and in the retinal pigment epithelium. In addition to these modifications, PEG conjugated with the BODYPI dye is used too, but LNPs based on this lipid have shown weaker reporter protein luminescence as compared to particles based on DMG-PEG2000 (1,2-dimyristoyl-*rac*-glycero-3-methoxypolyethylene glycol-2000), possibly because of lower pK_a_ of the BODYPI-PEG nanoparticles (∼5.0) in comparison with pK_a_ of the LNPs based on DMG-PEG2000: ∼6.5 (Xiong et al., 2020).

Thus, PEG, just as cholesterol, is necessary to stabilize a nanoparticle and to increase circulation time, but an excessive PEG content negatively affects properties of LNPs. Introduction of a polysarcosine (which has lower immunogenicity) instead of PEG may be promising.

### 3.4 RNA–LNP-based therapeutics: clinical trials

RNA–LNP-based therapeutics have become popular after successful use of two mRNA vaccines (RNA-1273 and BNT162b2) against SARS-CoV-2. To date, the number of approved mRNA vaccines has increased, but not all of them take advantage of LNPs for delivery (Hu et al., 2024). Aside from being used as vaccines, RNA–LNP constructs are employed to treat cancers (as encoded adjuvants, neoantigen vaccines, *in vivo* chimeric antigen receptor T-cell therapy [CAR-T], or epigenetic regulators) and hereditary diseases (as genetic editors) (Hu et al., 2024). On the ClinicalTrials.gov website, we searched for all clinical trials that have involved LNPs. In most studies, information about the delivery system is not disclosed, only 16 papers mention LNPs, and detailed composition is described in only five papers (Table 2). Lipid composition is often not disclosed by the companies that have developed the drug; hence, it is not possible to fully study the composition of the formulations in question. All known compositions from the above-mentioned clinical trials contain four lipids: an ionizable lipid, a phospholipid, cholesterol, and a PEGylated lipid, and the compositions themselves are already known (trials No. NCT03323398, CT05945485, and NCT05755620). Moderna utilizes a new ^13^C-labeled lipid, which is an analog of SM-102 and will allow for more accurate assessment of pharmacokinetics of mRNA–LNP therapeutics (Schellekens et al., 2011). Another new ionizable lipid, XH-07, is used by Duke University (NCT06468605). Side effects of the new lipids have not yet been investigated.

**TABLE 2:** mRNA–LNP therapeutics currently in clinical trials.

Indication	LNP composition	Vaccine type	Target	Phase	Clinical-Trials.gov identifier
Cancer	Not shown	mRNA	OX40L IL-23 IL-36γ	Phase 1	NCT03739931
Cancer	DLin-MC3-DMA, DSPC, Сholesterol, DSPE-PEG2000	mRNA	OX40L	Phase 1/2	NCT03323398
Intrahepatic cholangiocarcinoma	Not shown	srRNA	hepatocyte nuclear factor 4α (HNF4α)	Phase 1	NCT06572189
Glioma	XH-07, Сholesterol, DSPC, DMG-PEG 2000	srRNA	IL-12	Phase 1	NCT06468605
Hepatocellular carcinoma	Not shown	programmable epigenomic mRNA	Not shown	Phase 1/2	NCT05497453
HIV	Not shown	mRNA	Native-like HIV-1 envelope trimer	Phase 1	NCT05903339
Malaria	Not shown	RNA	*Plasmodium falciparum* circumsporozoite protein	Phase 1	NCT05581641
Malaria	Not shown	RNA	*P. falciparum* antigens	Phase 1/2	NCT06069544
Influenza	Not shown	mRNA	Influenza hemagglutinin	Phase 1	NCT05829356
Influenza	Ionizable lipid, DSPC, cholesterol, PEG lipid	mRNA	Full-length H1 HA of influenza A	Phase 1	NCT05945485
Influenza	ALC-307, DSPC, cholesterol, ALC-0159	mRNA	Hemagglutinin (HA)	Phase 1	NCT05755620
Cystic fibrosis	Not shown	mRNA	Сystic fibrosis transmembrane conductance regulator protein	Phase 1	NCT05712538
Ornithine transcarbamylase deficiency	Not shown	mRNA	Ornithine transcarbamylase	Phase 1/2	NCT04442347 NCT05526066 NCT06488313 NCT04416126
Ornithine transcarbamylase deficiency	Not shown	mRNA	Ornithine transcarbamylase	Phase 1/2	NCT03767270
Glucose-6-phosphatase (G6Pase-α) deficiency	O-11480, DPPC, cholesterol, DMG-PEG 2000	mRNA	G6Pase-α	Phase 1/2	NCT05095727
Hereditary transthyretin amyloidosis with polyneuropathy	Not shown	mRNA	CRISPR/Cas9	Phase 1	NCT04601051

Cancer vaccines have involved mRNAs encoding cytokines IL-23, IL-36γ, IL-12, or OX40L (NCT03739931, NCT03323398, and NCT06468605) as well as hepatocyte nuclear factor 4α (NCT06572189), which participates in hepatocyte proliferation. Linear mRNA-based vaccines are also being tested against infectious diseases such as influenza (NCT05829356 and NCT05755620), malaria (NCT06069544 and NCT05581641), and HIV (a booster vaccine; NCT05903339). Separately, drugs are being developed against hereditary disorders, including orphan diseases such as cystic fibrosis (NCT05712538), ornithine transcarbamylase deficiency (NCT04442347, NCT05526066, NCT06488313, NCT04416126, and NCT03767270), glucose-6-phosphatase deficiency (NCT05095727), and hereditary transthyretin amyloidosis with polyneuropathy (NCT04601051).

## 4 Development of novel lipids and novel approaches to targeted delivery

### 4.1 Creation of novel lipids

When new cationic/ionizable lipids are designed, addition of new functional groups or replacement of existing ones may not affect the biodistribution of nanoparticles within the body. Typical biodistribution with subsequent expression in the liver and spleen is characteristic of LNPs containing i) cationic/ionizable lipids linked through disulfide bonds and carrying an unsaturated tail and amine head (Shen et al., 2023; Lee et al., 2020), ii) lipids with tertiary-amine headgroups modified with a terminal hydroxyl group, ester bond, and branched saturated tails (Long et al., 2023), iii) modified aminoglycosides that are synthesized from natural existing aminoglycosides coupled with alkyl epoxides and acrylates (Yu et al., 2020), or iv) a cationic lipid carrying an amine head with two unsaturated tails (Hashiba et al., 2020).

During the construction of new delivery systems based on four-component LNPs, researchers try to synthesize new ionizable lipids having unique properties. As mentioned above, targeting of LNPs to desired organs can be achieved by means of ionizable lipids and/or via introduction of an additional helper lipid. For example, after intravenous injection of LNPs, positively charged DOTAP as a helper lipid ensures delivery to lungs (Dilliard et al., 2021).

For new LNP compositions, we evaluated the influence of chemical modifications of a cationic lipid on biodistribution and transfection efficiency (Tables 3–5), then analyzed in detail the distribution of an mRNA or protein product among organs and cell populations as well as methods and doses of administration, chemical and physical properties of the LNPs based on new lipids, and strategies for the design of new lipids (Table 6).

**TABLE 3:** Specific targeting of LNPs based on newly developed ionizable and helper lipids.

Molar proportion of lipids used, %	Development strategy	Top lipid	Administration	Distribution or expression of mRNA in targeted and other organs	Distribution among cell populations	Detection	Physico-chemical properties of particles	Reference
26.5 244cis 20 DOTAP 52 Cholesterol 1.5 C16-PEG2000	Replacement of saturated bonds with unsaturated ones in the lipid tail of an ionizable lipid	244cis 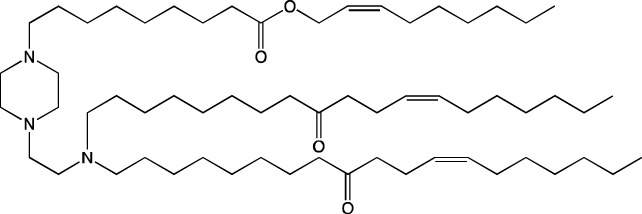	Intravenous retro-orbital FLuc mRNA 0.2 mg/kg	Lungs ∼ 80 % Spleen ∼ 15% Liver ∼ 5%		IVIS 6 h	Size 81.0 nm PDI 0.09 Zeta −3.7 mV EE 87.2%	Kim et al. (2023)
			Intravenous retro-orbital Cre mRNA 0.3 mg/kg	Lungs Liver Spleen	Healthy mouse: Endothelial cells 84.3% Fibroblasts 21.6% Immune cells 18.6% Epithelial cells 25.4% Pulmonary fibrosis: Endothelial cells 81.9% Fibroblasts 24.7% Immune cells 30.1% Epithelial cells 33.1%	IVIS 72 h		
30 RCB 4-8 39 DOTAP 30 Cholesterol 1 DMG-PEG2000	Selection of different ionizable groups for the amine head and alcohols for the lipid tail of an ionizable lipid	RCB 4-8 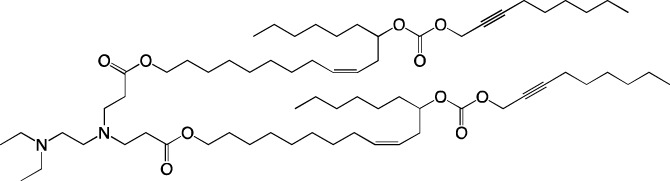	Intratracheal FLuc mRNA 0.125 mg/kg 0.25 mg/kg 0.5 mg/kg	Lungs Other organs n.d.	—	IVIS 6 h	Size 85.7 nm PDI 0.11 Zeta 9.7 mV EE 87.1%	Li B. et al. (2023)
73 29d 12 DOPE 9 Cholesterol 2 DMG-PEG2000	Screening of lengths, saturation levels, positions of a lipid tail, and of substituents in the amine head, selection of a helper lipid	29d 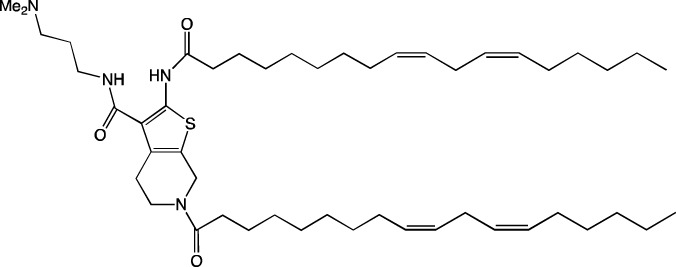	Intravenous FLuc mRNA 5 μg per mouse	Lungs Spleen Liver	—	IVIS 24 h	Size ∼75 nm PDI ∼ 0.2 EE ∼98.0%	Eygeris et al. (2024)
61 29d 11 DOPE 25 Cholesterol 3 DMG-PEG2000	Screening of lengths, saturation levels, positions of a lipid tail and of substituents in the amine head, selection of a helper lipid	29d 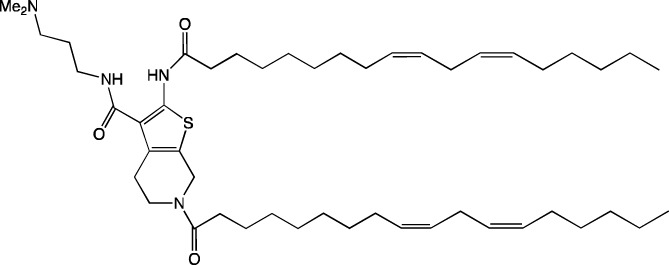	Intravenous FLuc mRNA 5 μg per mouse	Spleen Lungs Liver	—	IVIS 24 h	Size ∼ 75 nm PDI <0.3 EE ∼ 98.0%	Eygeris et al. (2024)
60 20b 20 DSPC 19 Cholesterol 1 DMG-PEG2000	Screening of lengths, saturation levels, positions of a lipid tail and of substituents in the amine head, selection of a helper lipid	20b 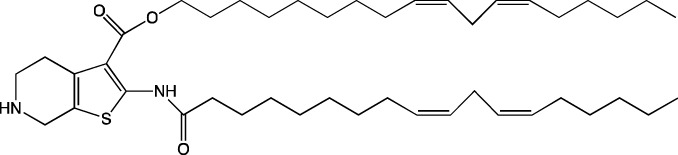	Intravenous Cre mRNA 0.3 μg per mouse	Eyes Other organs n.d.	Retinal pigment epithelium Photoreceptors Müller glia	IHC 96 h	EE ∼ 98.0%	Eygeris et al. (2024)
15-26 cKK-E15 25-55 Phospholipid with adamantyl group 28-46.5 Cholesterol or β-Cholesterol or 20α-OH Cholesterol 1.5-2.5 DMG-PEG2000	Addition of an adamantyl group; Variation of tail length and saturation	—	Intravenous Cre mRNA 1.0 mg/kg	Liver Spleen Bone Marrow	Liver: Kupffer cells ∼55% Endothelial cells ∼40% Hepatocytes ∼10% Spleen: Endothelial cells <5% Dendritic cells <5% Epithelial cells <5% Bone marrow: Hematopoietic cells <5%	FACS 3 day	Size 80–120 nm	Gan et al. (2020)
50 306-N16B 10 DOPC 38.5 Cholesterol 1.5 DMG-PEG2000	Selection of various amine cores and alkyl tail lengths	306-N16B 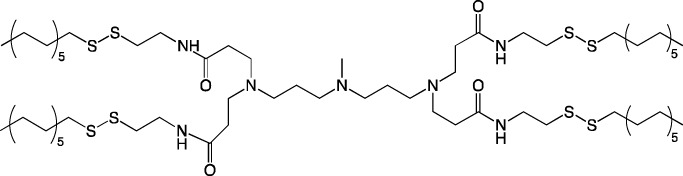	Intravenous Cre mRNA 0.75 mg/kg	Lungs ∼ 100% Liver n.d. Spleen n.d. Kidneys n.d.	Endothelial cells 35% Epithelial cells ∼ 0% Macrophages ∼ 0%	IVIS 7 day FACS 7 day	Size 81 nm PDI 0.20 Zeta −0.5 mV	Qiu et al. (2022)
25 L319 25 F-L319 10 DSPC 38.5 Cholesterol 1.5 DMG-PEG2000	Mixing of two ionizable lipids to obtain a five-component nanoparticle	L319 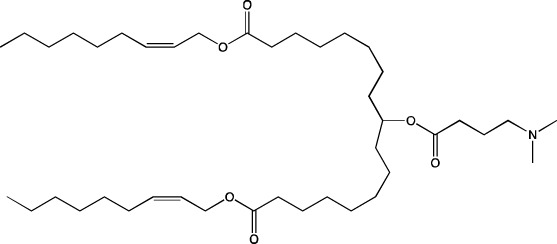 F-L319 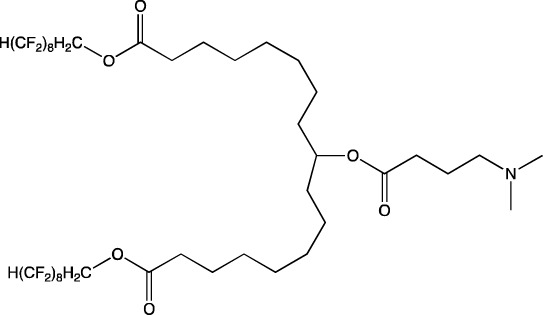	Intravenous FLuc mRNA 2 μg per mouse	Spleen ∼ 100% Liver n.d. Lungs n.d. Heart n.d. Kidneys n.d.	—	IVIS 24 h	Size 180 nm PDI 0.1 Zeta −4 mV EE 75.0%	Huo et al. (2023)
55 9A1P9 30 DOPE 40 Cholesterol 0.4 DMG-PEG2000	Screening of a panel of lipids differing in the number of zwitterionic atoms, alkyl tails, and tail length. Using a phospholipid instead of a cationic lipid	9A1P9 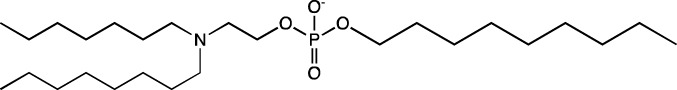	Intravenous FLuc mRNA 0.25 mg/kg	Spleen >90 % Liver <10% Lungs n.d. Heart n.d. Kidneys n.d.		IVIS 6 h	Size ∼ 150 nm PDI ∼ 0.13 Zeta ∼ −6 mV	Liu et al. (2021)
			Intravenous cre mRNA 0.25 mg/kg		Macrophages 30% B cells 6%	FACS 48 h		
30 5A2-SC8 25 9A1P9 30 Cholesterol 1 DMG-PEG2000	Screening of a panel of lipids differing in the number of zwitterionic atoms, alkyl tails, and tail length	9A1P9 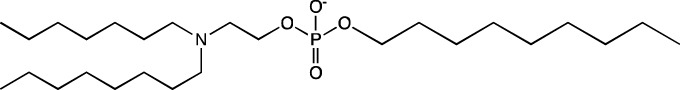	Intravenous FLuc mRNA 0.25 mg/kg	Liver ∼ 100% Spleen n.d. Lungs n.d. Heart n.d. Kidneys n.d.		IVIS 6 h	Size ∼ 260 nm PDI ∼ 2.5 Zeta ∼ −4 mV	Liu et al. (2021)
			Intravenous Cre mRNA 0.25 mg/kg		Hepatocytes 91%	FACS 48 h		
60 9A1P9 30 DDAB 40 Cholesterol 0.4 DMG- PEG2000	Screening a panel of lipids differing in the number of zwitterionic atoms, alkyl tails, and tail length. Using a phospholipid instead of a cationic lipid	9A1P9 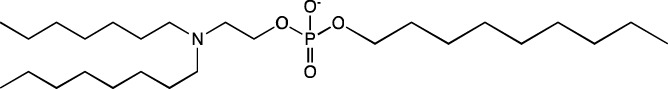	Intravenous mRNA 0.25 mg/kg	Lungs ∼ 100% Liver n.d. Spleen n.d. Heart n.d. Kidneys n.d.		IVIS 6 h	Size ∼ 150 nm PDI ∼ 0.08 Zeta ∼ −2.5 mV	Liu et al. (2021)
			Intravenous Cre mRNA 0.25 mg/kg		Endothelial cells 34% Epithelial cells 20% Immune cells 13%	FACS 48 h		
60 9A1P9 30 DOTAP 40 Cholesterol 0.4 DMG-PEG2000	Screening a panel of lipids differing in the number of zwitterionic atoms, alkyl tails, and tail length. Using a phospholipid instead of a cationic lipid	9A1P9 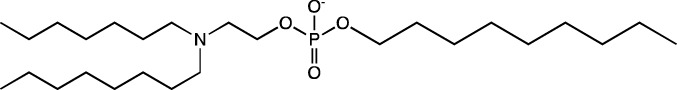	Intravenous FLuc mRNA 0.25 mg/kg	Lungs >90 % Spleen <10% Liver n.d. Heart n.d. Kidneys n.d.		IVIS 6 h	Size ∼ 150 nm PDI 0.1 Zeta ∼ −2 mV	Liu et al. (2021)
			Intravenous Cre mRNA 0.25 mg/kg			FACS 48 h		
35 Lipid 306O_i10_ 16 DOPE 46.5 Cholesterol 2.5 DMG-PEG2000	Screening a panel of lipids with different acrylamide tails and branching	306O_i10_ 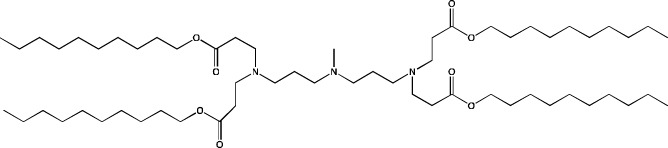	Intravenous Су5-labeled mRNA 0.75 mg/kg	Pancreas <1% Liver ∼ 100 % Spleen <1% Lungs n.d. Kidneys n.d. Heart n.d.	—	IVIS 1 h	—	Hajj et al. (2019)
			Intravenous FLuc mRNA 0.75 mg/kg	Pancreas <1% Liver ∼ 65 % Spleen ∼ 10% Lungs ∼ 15% Kidneys ∼10% Heart n.d.		IVIS 3 h		
35 Lipid 306O_i10_ 16 DOPE 46.5 Cholesterol 2.5 DMG-PEG2000	—	306O_i10_ 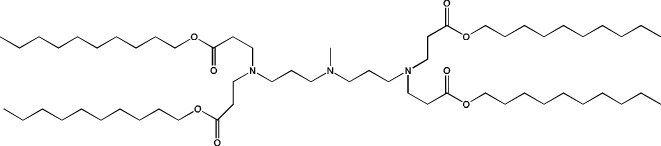	Intravenous Cre mRNA 2.0 mg/kg	Liver ∼ 95% Spleen ∼ 5% Kidneys <1% Heart n.d. Lungs n.d. Pancreas n.d.	Hepatocytes ∼ 70% Kupffer cells ∼ 15% Endothelial cells ∼ 15%	FACS 24 h	Size 124.6 nm PDI 0.12 Zeta 0.43 mV pK_a_ 6.4 EE 91.1%	Hajj et al. (2020)
35 Lipid 306O_i10_ 40 DOTAP 22.5 Cholesterol 2.5 DMG-PEG2000	Comparison of three lipids distinguished by their amine core and tails Comparison of different helper lipids	306O_i10_ 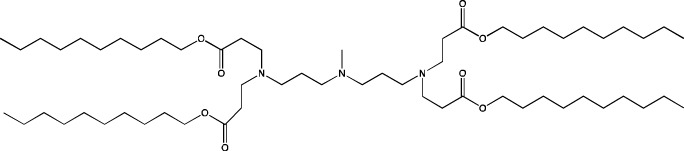	Intraperitoneal FLuc mRNA 0.5 mg/kg	Pancreas >90% Spleen <5% Liver <5% Lungs n.d.		IVIS 3 h	—	Melamed et al. (2023)
			Intraperitoneal Cy5-labeled mRNA 0.5 mg/kg		B cells 100% T cells ∼100% Macrophages ∼100%	FACS Instantly		

**TABLE 4:** Specific targeting of LNPs to placenta and tumors.

Molar proportion of lipids used, %	Development strategy	Top lipid	Administration	Distribution or expression of mRNA in targeted and other organs	Distribution among cell populations	Detection	Physico-chemical properties of particles	Reference
35 Lipid 306O_i10_ 16 DOPE 46.5 Cholesterol 2.5 DMG-PEG2000	Screening for various lipids	306O_i10_ 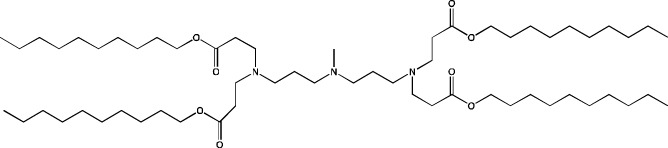	Intraperitoneal Cy5-labeled mRNA 0.5 mg/kg	Placenta Liver Spleen	—	IVIS 4 h	Size ∼ 120 nm PDI 0.1 EE ∼ 98.0%	Chaudhary et al. (2024)
			Intraperitoneal Cre mRNA 0.5 mg/kg		Trophoblasts 4.65% Immune cells 3.63% CD4^+^ cells 2% CD8^+^ cells 1% NK cells 3% Dendritic cells 1% Macrophages 2% Endothelial cells 4.19%	FACS 3 days		
20 DAL 30 DOPE 40 Cholesterol 0.75 DMG-PEG2000	Screening of various cyclic and noncyclic heads of an ionizable lipid	DAL4 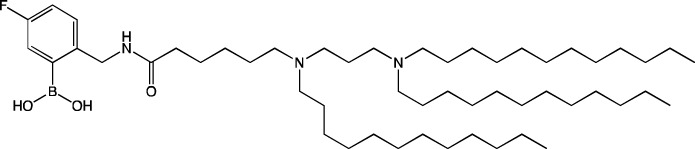	Intratumoral GFP mRNA 10 μg per injection	Tumor Other organs n.d.	CD45^+^ cells 31.9% CD11b^+^ cells 2.2% F4/80^+^ cells 3.29% CD3^+^ cells 0.82% CD19^+^ cells 98.2%	FACS 12 h	Size ∼ 150 nm PDI <0.1	Liu et al. (2022)
45 Ionizable lipid 10 DOPE 42.5 Cholesterol 2.5 DMG-PEG2000	Sequential optimization of the amino head group, tail group, linker, and substituent on an ionizable atom	A2-Iso5-2DC18 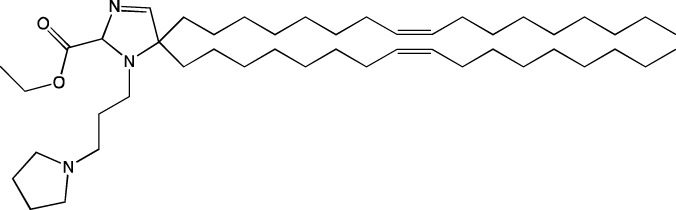 A12-Iso5-2DC18 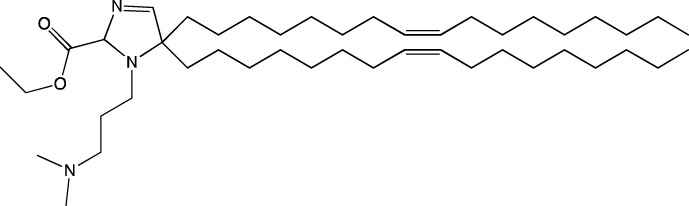	Subcutaneous FLuc mRNA 0.1 mg/kg	Injection site Lymph node		IVIS 5 h	—	Miao et al. (2019)
			Subcutaneous Cre mRNA 0.5 mg/kg		Macrophages 15% Dendritic cells 14% Leukocytes <3% Neutrophils <3%	FACS 48 h		
n.s. 4A3-SC8 n.s. DOPE n.s. Cholesterol n.s. PEG20005d	Choosing various ionizable groups for the amine head in the synthesis of a 4A3-SC8 analog Modification of PEG by BODIPY dyes using different linkers	PEG20005d 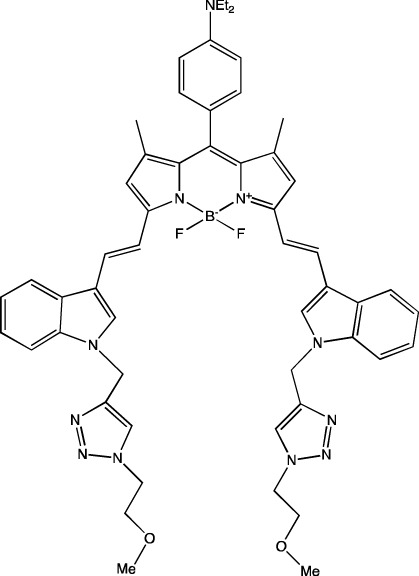	Subcutaneous FLuc mRNA (+ BODIPY fluorescent dye) 0.05 mg/kg	Tumor Liver Kidneys Pancreas	—	IVIS 6 h	—	Xiong et al. (2020)
n.s. A4 n.s. DOPE n.s. Cholesterol n.s. DMG-PEG2000	Testing of combinations of different epoxy cores and polyamide cores in the development of a new ionizable lipid	A4 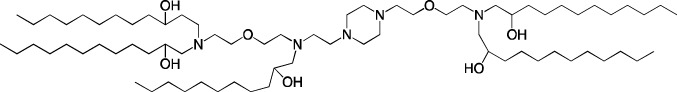	Intravenous FLuc mRNA 0.6 mg/kg	Nonpregnant mice: Uterus Pregnant mice: Placenta Liver Spleen Fetus n.d.		IVIS 6 h	Size ∼ 100 nm PDI <0.1 pKa ∼ 6.5 EE ∼ 90.0%	Swingle et al. (2023)
			Intravenous mCherry mRNA 1 mg/kg		Trophoblasts ∼6% Endothelial cells ∼ 3% Immune cells ∼ 5%	FACS 12 h		
35 С12-200 16 DOPE 46.5 Cholesterol 2.5 DMPE-PEG2000	—	—	Intravenous FLuc mRNA 0.5 mg/kg	Placenta traces Liver lion’s share Spleen Lungs traces Liver traces Heart n.d. Ovaries n.d.	—	IVIS 4 h	Size 110.2 nm PDI <0.139 pKa ∼ 5.619 EE 64.0%	Young et al. (2022)
				Liver ∼ 55% Spleen ∼ 40 % Lungs <2% Kidneys <1% Pancreas <1% Heart <1% Ovaries <1%		IVIS 4 h		

**TABLE 5:** Specific targeting of LNPs with variation of the number of lipids in composition and other development strategies.

60 CAP2-4 30 DOPE 0.75 DMG-PEG2000	Combination of an ionizable lipid with cholesterol. Testing different amine head structures	CAP2-4 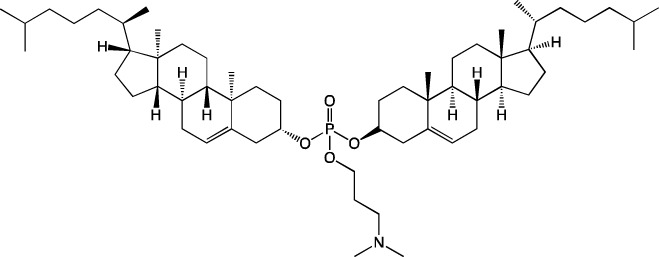	FLuc mRNA 7 μg per testis	Seminiferous tubules Other organs n.d.	—	IHC 24 h	Size 126.4 nm PDI 0.24 EE 80.0%	Du et al. (2023)
50 tB-UC18 50 DOPE	Screening of various alkyl saturated and cyclic substituents in para-substituted and tri-substituted benzaldehyde molecules	tB-UC18 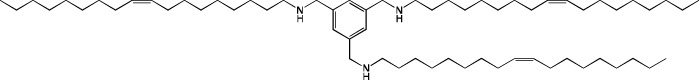	Intravenous FLuc mRNA (+DiD dye) 0.5 mg/kg	Spleen 95% Liver <5% Lungs n.d. Kidneys n.d. Heart n.d.	B cells 5% T cells ∼5% Macrophages ∼ 30% Dendritic cells 25%	IVIS 4 h FACS 4 h	Size 290 nm Zeta −25 mV	Li et al. (2021)
30 Vc Lipid 30 DOPE 50 Cholesterol	Addition of an alkyl radical with tertiary nitrogen atoms to various vitamins	Vc lipid 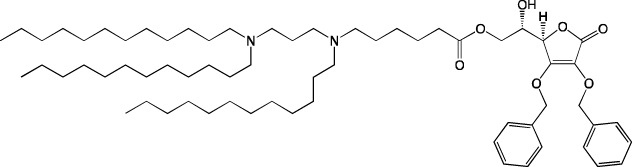	—	Macrophage lysosomes Other organs n.d.	Primary macrophages	—	Size 180 nm PDI 0.1 Zeta 30 mV EE 90.0%	Hou et al. (2020b)
n.s. R9H6 n.s. DOTAP	Switching the formulation order to a two-step procedure. Screening of various short peptides containing arginine	R9H6 amino sequence	Intratumoral NIS mRNA 350 µg/kg		Tumor 0 h 6.48% 12 h 2.67% 24 h 0.01%	FACS 0, 12, 24 h	Size 116.8 nm PDI 0.16	Blakney et al. (2019)
			Intratumoral NIS mRNA 350 µg/kg	Thyroid cancer Not measured in other organs		SPECT/CT 24 h		
35 Cephalin 16 DOPE 49 Cholesterol	Screening of cationic, ionizable, and zwitterionic lipids and of their ratios	Cephalin 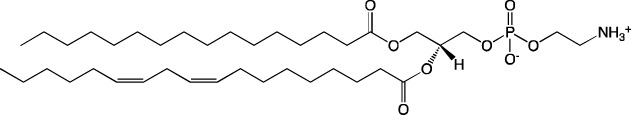	Intradermal eGFP small activating RNA 2 μg per explant	Skin explant Other organs n.d.	2.5% GFP-positive cells: Epithelial cells 40% Dendritic cells 16.2% Langerhans cells 12.0% Monocytes 9.6% Leukocytes 7.9% Fibroblasts 6.2% T cells 4.1% NK cells 3.6%	FACS 3 d	Size ∼ 370 nm Zeta −3 mV	Li Q. et al. (2023)

**TABLE 6:** Successful strategies for lipid chemical modification.

Graphical presentation	Description	Transfection effect	Targeting effect
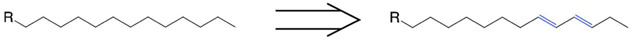	Changing the saturation of lipid tail Kim et al. (2023); Eygeris et al. (2024); Sarode et al. (2022)	↑ Transfection for unsaturated tails compared to saturated tails ↑ Transfection for saturated and double-bonded lipids compared to triple-bonded lipids	
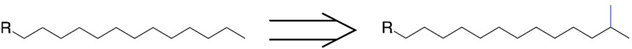	Using branched tails instead of unbranched tails Melamed et al. (2023); Shiraishi and Yokoyama (2019)	↑ Transfection for lipids containing an additional methyl group	
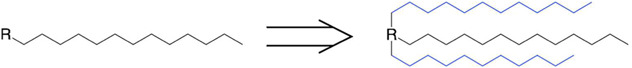	Changing the amount of a lipid tail Tenchov et al. (2021); Yu et al. (2020); Long et al. (2023)	↑ Transfection for two lipid tails instead of one or three lipid tails	
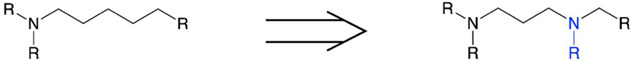	Changing the number of ionizable atoms Long et al. (2023); Kim et al. (2023)	↑ Transfection for a single inonizable zwitterion compared to multiple inonizable zwitterions ↑ Transfection for molecules with one ionizable group compared to two	
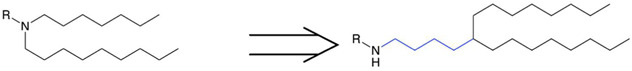	Adding a linker between an amine head and lipid tail Kim et al. (2023); Tenchov et al. (2021)	↑Transfection for ethylisocyanides	
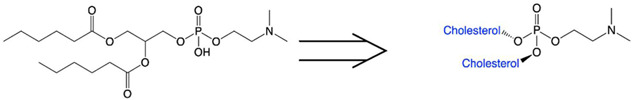	Combination of two lipids Du et al. (2023)	↑Transfection for dicholesterol with a tri-substituted ionizable group compared to MC3 and ALC-0315	
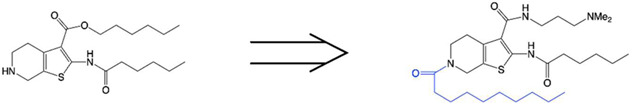	Addition of a spacer between the thiophene core (containing a lipid tail) and the ionizable group Tenchov et al. (2021)	↑Transfection with increasing spacer length, as a result of ↑pKa	
	Screening of various aliphatic and aromatic amine heads Sarode et al. (2022); Xiong et al. (2020); Liu et al. (2022); Long et al. (2023); Melamed et al. (2023); Hu et al. (2024)	↑Transfection for propylpiperazines ↑Transfection for the fluoroboryl substituent on the ionizable group ↑Transfection for mono-, di-, and tri-substituted amines relative to other functional groups ↑Transfection for amine head 306 compared to amine heads 113, 304, 200, and 504 ↑Transfection for quaternary ionizable amines, compared to tertiary ones in the case of a polymer molecule	
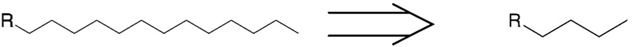	Changing the length of a lipid tail Shen et al. (2023); Long et al. (2023); Hu et al. (2024); Hou et al. (2020b)	↑ Transfection for 10–14 carbons in the lipid tail ↑ Transfection for 16 atoms in the lipid tail (C + S) compared to 12 and 14 atoms ↑ Transfection for 11–22 atoms in the lipid tail (C + S) compared to other tails	↑ Tropism to the spleen for 14–16 carbons in the lipid tail ↑ Tropism to the liver for the 9–10 carbons in the lipid tail
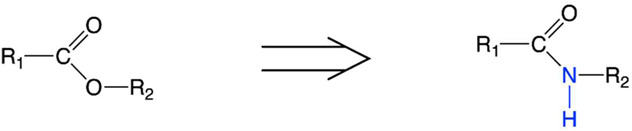	Replacement of an ester bond with an amide bond in a lipidoid molecule Hu et al. (2024)		↑ Tropism to the lungs ↓ Tropism to the liver

#### 4.1.1 Development of novel ionizable lipids

Some authors (Ni et al., 2022) have examined the distribution—among liver cells—of piperazine PPZ-A10–based LNPs with the dTomato reporter in Ai14 mice. The proportions of dTomato-positive cells in Ai14 mice were as follows: 60% of Kupffer cells, 55% of spleen macrophages, 35% of spleen dendritic cells, 30% of hepatocytes, 20% of liver dendritic cells, 25% of remaining spleen immune cells, 15% of remaining liver immune cells, and 10% of liver enterochromaffin cells (Ni et al., 2022); this pattern can serve as a good illustration for predicting reporter protein expression when four-component LNPs are used. The use of a new piperazine-based cationic lipid with unsaturated hydrophobic tails together with DOTAP in LNPs (Kim et al., 2023) affects the efficiency of delivery of reporter mRNA to lungs (as compared to SM-102 with saturated tails) by increasing the number of tdTomato-expressing cells in healthy mice and mice with induced pulmonary fibrosis. The tdTomato expression was detectable in endothelial cells, fibroblasts, immune cells, and epithelial cells. Notably, the use of LNPs based on another positively charged lipid, EPC, as a helper lipid significantly weakened the expression of the target protein in the lungs. The key role of DOTAP in the distribution to lungs is evident after intratracheal administration too (Li B. et al., 2023). Namely, replacement of DOTAP with DOPE in LNPs containing ionizable lipid RCB 4-8 (which consists of nonynyloxy tails linked to the amine center through an ester bond by an unsaturated branched alkyl linker) lowered reporter protein bioluminescence by almost half in lungs when nanoparticles based on the new lipid were administered.

A comprehensive study on new thiophene-based lipids, which have been used as ionizable lipids, was conducted not so long ago (Eygeris et al., 2024). During the development of the new lipids, those authors analyzed the length and saturation of the tail, an effect of restricting the conformation of the amino group by a piperidine ring, and the influence of pK_a_ as a consequence of introduction of a spacer between the piperidine ring and the ionizable head group. Most modifications did not affect the biodistribution of LNPs. Nonetheless, LNPs based on unsaturated lipids were better at encapsulating mRNA. The restriction of the amino group conformation by the piperidine ring influenced the yield of the target lipid during its synthesis. Finally, those authors noticed that extending the spacer improved transfection efficiency. By moving the unsaturated tails of lipids from the *ortho* position to the *para* position on opposite sides of the thiophene core and by modifying the ester so that it carries an ionizable head group, they were able to create a lipid called 29d, which as a component of LNPs, provided targeting to the spleen/lungs (Eygeris et al., 2024). Moreover, the ratio of delivery of LNPs between the lungs and spleen changed when the researchers varied the lipids that constitute the four-component particle (Eygeris et al., 2024). Using DOPE instead of DSPC and reducing the cholesterol content allowed to diminish the delivery of the LNPs into the liver compared to other organs.

Whitehead’s research group has been developing an ionizable lipid directly for delivery to the pancreas. Hajj et al. (2019) describes testing of ionizable lipids each containing two tertiary amines with acrylate tails of different lengths. The acrylate tail length had little effect on organ targeting, whereas branching improved delivery to all organs, including the pancreas. Those authors believe that these effects are likely due to the fact that the additional side group extends the distance between lipid molecules in the matrix of LNPs, thereby leading to better protonation of tertiary nitrogens and better packaging of mRNA. Another explanation is a hindrance of rotational mobility of branched lipids, with a consequent increase in the microviscosity of LNPs’ matrix and their stability, resulting in smaller particles with a narrower size distribution and increasing mRNA encapsulation (Hashiba et al., 2022). For LNPs based on the most effective lipid, 306O_i10_, switching the route of administration from intraperitoneal to intravenous caused the same particles to lose the ability to ensure expression of a luminescent product in the pancreas and the ability to accumulate in minimal quantities in lungs and the heart; LNPs were still present only in the liver and spleen. It is worth noting that expression of the desired product in the pancreas and other organs relative to expression in the liver was negligible even for the most effective lipid composition (Hajj et al., 2019). LNPs based on a lipid called 306O_i10_ and cationic helper lipid DOTAP are able to deliver reporter mRNA into pancreatic β-cells. Presumably, delivery to the pancreas via intraperitoneal administration is mediated by an interaction of cationic particles with negatively charged components of the peri-islet membrane, which contains laminins, type IV collagen, and perlecan. Macrophages alone are responsible for LNP delivery specifically to β-islets and absorb LNPs and then secrete extracellular vesicles, which again transfect β-cells in the islets. This hypothesis is supported by the finding that other cationic helper lipids (EPC and DDAB) within such LNPs cause similar protein expression in the pancreas (Melamed et al., 2023). For the LNPs based on lipid 306O_i10_, the distribution among different types of liver cells has been assessed too (Hajj et al., 2020). The expression of the reporter protein proved to be distributed among hepatocytes, Kupffer cells, and endothelial cells at a 70:15:15 ratio.

Another area of research is assessment of a distribution of LNPs based on ionizable lipids containing an amino head group and an acrylamide tail with a disulfide bond (Qiu et al., 2022). *In vivo* efficiency of luciferase synthesis (by translation) when four-component LNPs were employed improved with increasing acrylamide tail length, and all the nanoparticles got distributed to lungs. After replacement of helper lipid DOPC (1,2-dioleoyl-*sn*-glycero-3-phosphocholine) with either DSPC or DOPE, the lung distribution persisted for LNPs based on the most effective lipid: 306-N16B. Of note, LNPs based on a similar ionizable lipid, 306-O12B (containing an ester bond instead of an amide bond) got completely distributed to the liver. In an analysis of the protein corona of LNPs based on either lipid 306-N16B or lipid 306-O12B, it was found that the LNPs targeted to the liver contain albumin, ApoE, and complement component C1 in the protein corona at 9%, 5%, and 5%, respectively, of the total corona proteins, whereas LNPs targeted to lungs contain albumin, fibrinogen β-chain, and fibrinogen γ-chain at 17%, 5%, and 5%, respectively, of the total corona proteins. It was also revealed that negatively charged proteins predominantly (80%) constitute the protein corona of the LNPs targeted to the lungs. LNPs based on the most effective lipids (306-N16B or 113-N16B) with different amine cores ensured the expression of a luminescent reporter protein exclusively in the lungs, mainly in endothelial cells with little expression in macrophages.

In another study (Huo et al., 2023), a new lipid was synthesized, F-L319, which is a fluorine-containing version of lipid L319, which in turn is a biodegradable modification of MC3 owing to ester bonds in alkyl chains. The fluorinated version of the lipid was chosen due to higher stability of nanoparticles based on it. After injection, four-component LNPs based on either L319 or F-L319 resulted in the expression of a luminescent product exclusively in the spleen, with the particles based on the fluorinated lipid resulting in less luminescence as compared to the nonfluorinated ones. Unexpectedly, mixing of the two ionizable lipids in five-component LNPs enhanced the expression of the reporter protein without a change in LNP biodistribution.

Altogether, the *in vivo* findings in animals indicate that lipid compositions containing novel ionizable lipids can increase tropism to various organs such as the pancreas, lungs, and liver. Thus, the development of new ionizable lipids is an effective way to improve targeted delivery of LNPs to certain organs.

#### 4.1.2 Construction of novel helper lipids

The design of new helper lipids has been less active than the development of new ionizable lipids. In Gan et al. (2020), aside from the standard strategy of trying different tail lengths and tail saturation levels, an adamantyl group was added to a phospholipid molecule that is used to improve the solubility of compounds and to impede the access of proteolytic enzymes (Liu et al., 2011). No significant difference was found between distributions of four-component particles containing an ionizable lipid called cKK-E15 and various ratios of components; all reporter genes were expressed in the liver, with relatively low expression levels in the spleen and a distribution among Kupffer cells, endothelial cells, and hepatocytes in descending order of intensity (Gan et al., 2020). Researchers have done a lot of work on the synthesis of new zwitterionic phospholipids (Liu et al., 2021). For the new helper lipids, the best solutions used for ionizable lipids have been copied: an ionizable amine group and an additional alkyl tail have been introduced. Alkyl substituents and a relatively small charged head group of lipids allow them to form an inverted hexagonal (HII) phase instead of a lamellar one when LNP components get incorporated into endosomal membranes; this phenomenon gives a more efficient release of encapsulated molecules from LNPs within endosomes (Du et al., 2023). The synthesized phospholipids (Liu et al., 2021) have had different lengths of the alkyl tail and various substituents on the ionizable amine group. Four-component LNPs containing phospholipids carrying one zwitterion with two hydrophobic tails containing 10–12 carbons in the ionizable amine group have turned out to be the best in terms of reporter protein expression in cellular systems. Final ionizable phospholipid 9A1P9 has acted as both an ionizable lipid and a helper lipid, thereby ensuring LNP delivery to the liver, spleen, or lungs when LNP composition contained ionizable lipid 5A2-SC8 or helper lipid DOPE or DDAB, respectively. *In vivo* data have also revealed a correlation between the length of the alkyl tail of the phosphorus group and the expression level of a reporter protein in the liver and spleen. The expression of luciferase is the highest with an alkyl tail length of 9–10 carbons for liver expression and 14–16 carbons for spleen expression (Liu et al., 2021).

Among strategies for the construction of new phospholipids, the only successful one is the addition of ionizable atoms to the molecule; this approach turns the molecule into a mixture of a helper and ionizable lipid. Other strategies for designing new phospholipids for LNP formulations are not conducive to targeting of the resulting LNPs to a specific organ, but perhaps the new phospholipids within LNPs will be able to increase overall efficiency of cell transfection.

### 4.2 Specific targeting

Separately, we would like to discuss such specific use cases of LNPs as delivery to the placenta and to a malignant tumor.

#### 4.2.1 Targeting to the placenta

Data on the biodistribution of four-component particles into the placenta or fetus during pregnancy are currently insufficient, but four-component LNPs based on commercial lipid C12-200 result in the expression of a reporter product in ovaries of nonpregnant mice (Swingle et al., 2023). In the work just cited, these particles served as controls for designing an ionizable lipid based on polyamide cores with epoxy tails for placenta targeting to treat placental diseases. LNPs based on lipids A4 and B5 along with helper lipid DOPE were able to induce the expression of a luminescent product in trophoblasts, endothelial cells, and immune cells within the placenta. In another article (Young et al., 2022), investigators also tested nanoparticles based on C12-200 along with the DOPE helper lipid. The most pronounced bioluminescence of the reporter protein was registered in the liver and spleen, and less than 1% of the expression was seen in ovaries, implying possible usefulness of these LNPs as a delivery system for the treatment of placental diseases.

Targeting of LNPs based on lipid 306O_i10_ in pregnant mice has been researched too (Chaudhary et al., 2024). There was accumulation of the luminescent product in the placenta but not in the embryos. No more than 5% of trophoblasts and immune and endothelial cells expressed the tdTomato reporter. Among immune cells, no more than 3% of leukocytes and no more than 2% of antigen-presenting cells expressed tdTomato (Chaudhary et al., 2024).

#### 4.2.2 Targeting to tumors

In some cases, for example, in cancer vaccines, there is no need to deliver mRNA to a target organ, and such a therapeutic agent can have an anticancer effect when administered intramuscularly (Xu et al., 2024). In the paper just cited, a cancer vaccine used in this way caused antigen-specific activation, which led to suppression of tumor growth and improved the survival of mice.

Malignant tumors are a promising target for standard four-component LNPs carrying new ionizable lipids because when the nanoparticles are administered, a malignant tumor is in the fourth place (after the liver, spleen, and blood) in terms of the presence of the LNPs (Kumar et al., 2023); this observation facilitates the creation of new lipid compositions for the targeting of malignant tumors. In an *in vivo* tumor model (Liu et al., 2022) (created via administration of B16F10 cells subcutaneously to C57BL/6 mice), after intratumoral injection of GFP mRNA within LNPs, 9% of cells expressed GFP at 12 h after the injection, with 32% of cells among them being immune cells, mainly B lymphocytes. Diamino lipid derivatives were employed there as the ionizable lipid, and the most effective of them contained an aromatic polar “head” and three unsaturated tails. Delivery of proinflammatory cytokines as an mRNA–LNP complex into a tumor may hold promise from the standpoint of cancer therapy. After intratumor injection, mRNAs of cytokines IL-12 and IL-27 within LNPs simultaneously reduced tumor size and increased the survival of mice, while enhancing tumor infiltration by CD45-positive leukocytes (Liu et al., 2022). In another murine cancer model (Xiong et al., 2020) (based on human breast cancer caused by SUM159 cells), four-component nanoparticles containing ionizable lipid 4A3-SC8 localized to the tumor and expressed a reporter protein at 6 h after subcutaneous administration.

A library of other ionizable lipids, consisting of various amine heads and alkyl tails linked by an isocyanide spacer, was screened in an anticancer study on mice (Miao et al., 2019). Results from the screening of more than 1,000 compositions of LNPs (carrying different ionizable lipids) on cells showed that protein expression is higher in nanoparticles containing ionizable lipids with long and unsaturated alkyl chains; the presence of an ester moiety in the ionizable lipid improved the efficiency of delivery, while the presence of a hydroxyl group worsened it. A second *in vivo* screening enabled the researchers to determine favorable structural features of the ionizable part of the lipid: 1) the presence of two amines separated by three carbons, 2) the absence of hydroxyl groups, and 3) the presence of at least one tertiary amine. When the LNPs were administered intraperitoneally, luciferase expression was detectable near the injection site and in an ipsilateral lymph node. In the lymph node, ∼15% of antigen-presenting cells expressed the reporter protein, but only 2% of lymphocytes. The LNPs were not administered intratumorally or intravenously; even a subcutaneous injection was enough to improve the survival rate of the mice with cancer.

### 4.3 Variation of the number of lipids in LNP composition

There are strategies for LNP design that differ from the standard four-component formulation. For example, lipid CAP-2 (cholesterol‐amino‐phosphate) has been created (Du et al., 2023), which combines an ionizable MC3 group and two molecules of cholesterol instead of alkyl tails, and this approach reduces the number of lipid components to three. When such an ionizable lipid was being designed, the layout of the amine core was altered, and alkyl substituents of different lengths and different linkers were utilized. During the construction of CAP molecules, the critical packing parameter, which characterizes the resultant phase during endosomal fusion, was calculated, and the molecules were designed in such a way that after the fusion, an inverted hexagonal (HII) phase formed, which is characterized by the greatest release of mRNA from the endosome. In animal trials, three-component LNPs containing CAP-2 have proven to be more effective at transfecting mouse seminiferous tubules as compared to four-component nanoparticles containing lipids MC3 and ALC-0315, which have not shown transfection activity after administration of LNPs based on them into testes. It is important to mention that other organs have not been studied, and therefore the question of specificity of such nanoparticles remains open (Du et al., 2023).

In another study (Yang et al., 2020), researchers assembled three-component nanoparticles in which they used lipid iBL0713, which combines functions of ionizable and helper lipids. For these LNPs, the biodistribution and expression of a reporter gene were evaluated by the IVIS (*in vivo* imaging system) method for the detection of luciferase and of Cy5-labeled mRNA encoding it, respectively. After detection of Cy5-labeled mRNA, its distribution was mainly seen in kidneys and to a lesser extent in the liver and spleen, while luciferase luminescence was detectable mainly in the liver and less in the spleen. Notably, Cy5-labeled mRNA was present in all organs, including the pancreas, brain, and thymus, where the reporter protein expression was not detectable (Yang et al., 2020).

LNPs made of two lipids—DOPE and a tri-substituted benzaldehyde derivative (Li et al., 2021)—have been found to cause the expression of a luminescent product exclusively in the spleen when administered intravenously but not subcutaneously or intramuscularly. To choose a final lipid, various alkyl saturated and cyclic substituents in *para*-substituted and tri-substituted benzaldehyde molecules were screened in that work, followed by selection of the most favorable ratio of the two lipids to mRNA. In tests on cultured cells, lipid-based particles with unsaturated tails manifested the greatest efficiency. The resulting nanoparticles were internalized mainly by macrophages and dendritic cells.

Three-component LNPs have been applied to antimicrobial treatments (Hou et al., 2020b). These LNPs contain DOPE, cholesterol, and vitamins modified by alkyl groups with tertiary amines; these compounds replace cationic lipids. To find the best lipid compositions, researchers have compared all the vitamin nanoparticles with each other. The greatest effectiveness was shown by composition with a lipid based on vitamin C (Hou et al., 2020b). Vitamin C–based nanoparticles containing antimicrobial peptide AMP-IB367 fused with cathepsin via a cathepsin-cleavable linker got delivered into macrophage lysosomes, thereby enabling the targeting of the payload to this cell compartment. The targeting was mediated not by the decoration of the particles but by the protein synthesized from mRNA directly in the cell. These LNPs were able to reduce the bacterial load and improve the survival rate of mice with sepsis.

Thus, proportions (%) of lipids within LNPs can vary. The minimal composition of effectively functioning LNPs includes an ionizable lipid and structural lipid, but three-component compositions of LNPs have been used successfully too. All these modifications of LNP composition may be interesting regarding the targeting of new tissues and cell types.

### 4.4 Other strategies

When nanoparticles are fabricated, a modified sequence of formulation is also utilized (Li Q. et al., 2023): mRNA is encapsulated into short peptides containing arginine, then this complex is formulated into cationic lipid DOTAP, resulting in the formation of particles with a separate membrane and core. In this procedure, different versions of arginine peptides contain stearyl, cholesterol, and various amino acids, but in cell assays, the best results have been shown by a peptide consisting of nine arginines and six histidines. After intraperitoneal administration of radioactive iodine and LNPs containing the mRNA of receptor NIS (which ensures the transfer of radioiodine into cancer cells), the localization of radioactive iodine exclusively in the thyroid gland was proven by computed tomography. The treatment reduced tumor size by a factor of five at 18 days after the induction as compared to control (Li Q. et al., 2023). Evidence from human skin explants administered intradermally (Blakney et al., 2019) indicates that the application of three-component LNPs containing a charged lipid and formulated together with DOPE and cholesterol can lead to high expression of a reporter protein. In that work, LNPs based on cationic lipids DDAB and DOTAP, ionizable lipid C12-200, or zwitterionic Cephalin manifested an ability to mediate the expression of small activating RNA in the human explants, for a maximum period of up to 21 days with DOTAP. Transfection of the largest number of cells was achieved by particles based on Cephalin. All compositions showed a similar distribution among cell types: ∼40% in the epithelium, ∼10% in fibroblasts, and ∼50% in immune cells (natural killers, leukocytes, T lymphocytes, dendritic cells, monocytes, and Langerhans cells).

To summarize, unconventional approaches to encapsulation of RNA into LNPs also ensure its efficient delivery. Rearranging the order of addition of lipids to mRNA and the use of peptides for mRNA encapsulation are new methods that may be suitable for the development of LNP compositions.

## 5 Concluding remarks and prospects

Improvements in RNA delivery systems based on LNPs yielded impressive results in the last few years. Nevertheless, the use of LNPs for targeted delivery to desired organs still requires optimization of lipid composition. In our review, we discussed successful examples of variations of lipid composition for targeted delivery to the spleen, lungs, pancreas, thyroid gland, placenta, and testes. Distribution of LNPs to the liver is the most typical after intravenous administration, especially for nanoparticles smaller than 100 nm. Through changes in lipid composition, it is possible to achieve preferential (in some cases, complete) redirection of nanoparticle accumulation to other organs. Apparent pK_a_ of LNPs plays an important role in this effect. For instance, nanoparticles having pK_a_ < 5 (they possess a negative charge in the bloodstream) tend to accumulate in the spleen, whereas particles with pK_a_ > 11 (positive charge) in lungs. A desired value of apparent pK_a_ of LNPs can be achieved by changing the helper lipid (SORT technology) and/or by using a combination of several ionizable cationic lipids. Application of bulkier lipids indirectly affects apparent pK_a_ of LNPs by altering the packing of lipids in the nanoparticle and thereby changing the accessibility of ionizable groups of lipids to protonation. Redirection of LNP accumulation to the spleen can be accomplished via reductions in the amount of “liver-specific” cholesterol in the nanoparticles. On the other hand, for some organs, such as eyes and the brain, effective delivery systems based on LNPs have not yet been devised, and in this case, delivery to the target organ is achieved only via direct administration of the therapeutic agent.

At present, computer technologies are actively utilized to design and improve LNPs: from computer modeling to machine learning and artificial intelligence (Du et al., 2023; Maharjan et al., 2023; Cornebise et al., 2021). During the development of new ionizable lipids, the most time-consuming task is the screening of large numbers of diverse LNPs. Application of artificial intelligence to improve predictions of properties of the resultant nanoparticles can reduce screening time (Maharjan et al., 2023). In Tilstra et al. (2023), to improve the structure of a cationic lipid, an iteration method was employed, where the structure was divided into separate parts, and then a step-by-step modification of each of them was performed. Because subsequent modifications did not cancel out effects of previous optimizations, each subsequent alteration of the structure of the cationic lipid led to an improvement of its properties. By this approach, optimal length of hydrocarbon chains, optimal numbers of tertiary nitrogen atoms and hydroxyl groups, and their optimal location inside the molecule were selected. With the help of this methodology, researchers successfully obtained LNPs with higher transfection efficiency and strong expression of a target protein (Tilstra et al., 2023).

Another important characteristic of LNPs is the efficiency of the endosomal release of mRNA. For this purpose, it is necessary that an ionizable lipid ensure the transition of the lamellar phase to an inverted hexagonal phase during endosomal fusion (Ramezanpour and Tieleman, 2022). In Cornebise et al. (2021), using molecular dynamics simulations, investigators were able not only to select new lipid structures but also to determine the parameters that affect their effectiveness. For example, it was demonstrated that in addition to Coulombic interactions with the phosphate group of RNA, cationic lipids can also interact with aromatic moieties of nitrogenous bases through π–π interaction as well as form hydrogen bonds between molecules. New lipids that fully implement all types of interactions with RNA indeed gave better experimental results on encapsulation and transfection (Cornebise et al., 2021).

Taken together, these data indicate that the development and implementation of machine learning and artificial intelligence technologies for predicting particle behavior are promising areas for LNP optimization. Successful application of these approaches to targeted delivery of LNPs requires interdisciplinary competencies in the fields of molecular genetics, physical chemistry, nano- and biotechnology, and bioinformatics.

An important aspect of the development of new LNP formulations is their safety. Some studies have shown that LNPs themselves can have strong inflammatory potential (Korzun et al., 2024; Ndeupen et al., 2021). The toxicity can be due to individual lipids (for example, activation of the complement system by PEG within LNPs) or to mRNA present in LNPs, which is able to activate innate immune-system components (Hou et al., 2021; Muslimov et al., 2023). Administration of mRNA–LNP constructs can induce liver and lung injuries or upregulate markers of activated microglia in rodents (Kirshina et al., 2022; Kedmi et al., 2010; Sedic et al., 2018). For cationic lipids that are present in LNPs, cytotoxicity can be caused by a quaternary ammonium group, whose mechanism of toxic action is mediated by a signaling pathway dependent on caspase activation and mitochondrial dysfunction (Cui et al., 2018). Notably, a similar mechanism of cytotoxic action is reported for mRNA–LNP therapeutics. After entering a lysosome, nanoparticles are hydrolyzed into “free ions,” which can pass through the nuclear membrane or penetrate into mitochondria, thereby forming reactive oxygen species, which can ultimately lead to DNA damage and apoptosis (Azarnezhad et al., 2020).

Another promising field of construction of LNPs for targeted delivery to organs is research into a combination of approaches to optimize lipid composition and into additional covalent modification of the LNP surface (Priya et al., 2023). Various modifications of LNPs with antibodies (Marques et al., 2023), peptides (Kim et al., 2024), aptamers (Liang et al., 2015), or sugars (Goswami et al., 2019) have proven to be effective at targeted delivery to certain types of cells. We believe that a combination of these approaches can give better characteristics of targeted delivery.

## References

[B1] Alvarez-Benedicto E. Farbiak L. Marquez Ramirez M. Wang X. Johnson L. T. Mian O. (2022). Optimization of phospholipid chemistry for improved lipid nanoparticle (LNP) delivery of messenger RNA (mRNA). Biomater. Sci. 10, 549–559. 10.1039/d1bm01454d 34904974 PMC9113778

[B2] Azarnezhad A. Samadian H. Jaymand M. Sobhani M. Ahmadi A. (2020). Toxicological profile of lipid-based nanostructures: are they considered as completely safe nanocarriers? Crit. Rev. Toxicol. 50, 148–176. 10.1080/10408444.2020.1719974 32053030

[B3] Billingsley M. M. Gong N. Mukalel A. J. Thatte A. S. El-Mayta R. Patel S. K. (2024). *In vivo* mRNA CAR T cell engineering via targeted ionizable lipid nanoparticles with extrahepatic tropism. Small 20, e2304378. 10.1002/smll.202304378 38072809

[B4] Birke A. Ling J. Barz M. (2018). Polysarcosine-containing copolymers: synthesis, characterization, self-assembly, and applications. Prog. Polym. Sci. 81, 163–208. 10.1016/j.progpolymsci.2018.01.002

[B5] Blakney A. K. McKay P. F. Ibarzo Y. B. Hunter J. E. Dex E. A. Shattock R. J. (2019). The skin you are in: design-of-experiments optimization of lipid nanoparticle self-amplifying RNA formulations in human skin explants. ACS Nano 13, 5920–5930. 10.1021/acsnano.9b01774 31046232 PMC7007275

[B6] Chaudhary N. Newby A. N. Arral M. L. Yerneni S. S. LoPresti S. T. Doerfler R. (2024). Lipid nanoparticle structure and delivery route during pregnancy dictate mRNA potency, immunogenicity, and maternal and fetal outcomes. Proc. Natl. Acad. Sci. U. S. A. 121, e2307810121. 10.1073/pnas.2307810121 38437545 PMC10945816

[B7] Chawla H. Vohra V. (2023). “Firms and the Welfare State: When, Why, and How Does Social Policy Matter to Employers?,” in StatPearls. Treasure Island, FL: StatPearls Publishing

[B8] Cheng Q. Wei T. Farbiak L. Johnson L. T. Dilliard S. A. Siegwart D. J. (2020). Selective organ targeting (SORT) nanoparticles for tissue-specific mRNA delivery and CRISPR-Cas gene editing. Nat. Nanotechnol. 15, 313–320. 10.1038/s41565-020-0669-6 32251383 PMC7735425

[B9] Cheng X. Lee R. J. (2016). The role of helper lipids in lipid nanoparticles (LNPs) designed for oligonucleotide delivery. Adv. Drug Deliv. Rev. 99, 129–137. 10.1016/j.addr.2016.01.022 26900977

[B10] Cornebise M. Narayanan E. Xia Y. Acosta E. Ci l Koch H. (2021). Discovery of a novel amino lipid that improves lipid nanoparticle performance through specific interactions with mRNA. Adv. Funct. Mater. 32. 10.1002/adfm.202106727

[B11] Cui S. Wang Y. Gong Y. Lin X. Zhao Y. Zhi D. (2018). Correlation of the cytotoxic effects of cationic lipids with their headgroups. Toxicol. Res. (Camb) 7, 473–479. 10.1039/c8tx00005k 30090597 PMC6062336

[B12] Da Silva Sanchez A. J. Dobrowolski C. Cristian A. Echeverri E. S. Zhao K. Hatit M. Z. C. (2022). Universal barcoding predicts *in vivo* ApoE-independent lipid nanoparticle delivery. Nano Lett. 22, 4822–4830. 10.1021/acs.nanolett.2c01133 35671473

[B13] Di J. Du Z. Wu K. Jin S. Wang X. Li T. (2022). Biodistribution and non-linear gene expression of mRNA LNPs affected by delivery route and particle size. Pharm. Res. 39, 105–114. 10.1007/s11095-022-03166-5 35080707 PMC8791091

[B14] Dilliard S. A. Cheng Q. Siegwart D. J. (2021). On the mechanism of tissue-specific mRNA delivery by selective organ targeting nanoparticles. Proc. Natl. Acad. Sci. U. S. A. 118, e2109256118. 10.1073/pnas.2109256118 34933999 PMC8719871

[B15] Du S. Li W. Zhang Y. Xue Y. Hou X. Yan J. (2023). Cholesterol-amino-phosphate (CAP) derived lipid nanoparticles for delivery of self-amplifying RNA and restoration of spermatogenesis in infertile mice. Adv. Sci. (Weinh) 10, e2300188. 10.1002/advs.202300188 36748274 PMC10104632

[B16] Evers M. Kulkarni J. van der Meel R. Cullis P. Vader P. Schiffelers R. M. (2018). State-of-the-Art design and rapid-mixing production techniques of lipid nanoparticles for nucleic acid delivery. Small Methods 2. 10.1002/smtd.201700375

[B17] Eygeris Y. Gupta M. Kim J. Jozic A. Gautam M. Renner J. (2024). Thiophene-based lipids for mRNA delivery to pulmonary and retinal tissues. Proc. Natl. Acad. Sci. U. S. A. 121, e2307813120. 10.1073/pnas.2307813120 38437570 PMC10945828

[B18] Gan Z. Lokugamage M. P. Hatit M. Z. C. Loughrey D. Paunovska K. Sato M. (2020). Nanoparticles containing constrained phospholipids deliver mRNA to liver immune cells *in vivo* without targeting ligands. Bioeng. Transl. Med. 5, e10161. 10.1002/btm2.10161 33758781 PMC7974401

[B19] Gao K. Li J. Song H. Han H. Wang Y. Yin B. (2023). *In utero* delivery of mRNA to the heart, diaphragm and muscle with lipid nanoparticles. Bioact. Mater 25, 387–398. 10.1016/j.bioactmat.2023.02.011 36844366 PMC9950423

[B20] Gautam M. Jozic A. Su G. L. Herrera-Barrera M. Curtis A. Arrizabalaga S. (2023). Lipid nanoparticles with PEG-variant surface modifications mediate genome editing in the mouse retina. Nat. Commun. 14, 6468. 10.1038/s41467-023-42189-3 37833442 PMC10575971

[B21] Gomi M. Sakurai Y. Sato M. Tanaka H. Miyatake Y. Fujiwara K. (2023). Delivering mRNA to secondary lymphoid tissues by phosphatidylserine-loaded lipid nanoparticles. Adv. Healthc. Mater 12, e2202528. 10.1002/adhm.202202528 36535635

[B22] Gong L. Zhang Y. Wang L. Zhao X. Qiu X. Yang X. (2024). Advancing vaccine development: evaluation of a mannose-modified lipid nanoparticle-based candidate for African swine fever p30 mRNA vaccine eliciting robust immune response in mice. Int. J. Biol. Macromol. 270, 132432. 10.1016/j.ijbiomac.2024.132432 38761609

[B23] Goswami R. Chatzikleanthous D. Lou G. Giusti F. Bonci A. Taccone M. (2019). Mannosylation of LNP results in improved potency for self-amplifying RNA (SAM) vaccines. ACS Infect. Dis. 5, 1546–1558. 10.1021/acsinfecdis.9b00084 31290323

[B24] Gueguen C. Ben Chimol T. Briand M. Renaud K. Seiler M. Ziesel M. (2024). Evaluating how cationic lipid affects mRNA-LNP physical properties and biodistribution. Eur. J. Pharm. Biopharm. 195, 114077. 10.1016/j.ejpb.2023.08.002 37579889

[B25] Hajj K. A. Ball R. L. Deluty S. B. Singh S. R. Strelkova D. Knapp C. M. (2019). Branched-tail lipid nanoparticles potently deliver mRNA *in vivo* due to enhanced ionization at endosomal pH. Small 15, e1805097. 10.1002/smll.201805097 30637934

[B26] Hajj K. A. Melamed J. R. Chaudhary N. Lamson N. G. Ball R. L. Yerneni S. S. (2020). A potent branched-tail lipid nanoparticle enables multiplexed mRNA delivery and gene editing *in vivo* . Nano Lett. 20, 5167–5175. 10.1021/acs.nanolett.0c00596 32496069 PMC7781386

[B27] Hald A. C. Kulkarni J. A. Witzigmann D. Lind M. Petersson K. Simonsen J. B. (2022). The role of lipid components in lipid nanoparticles for vaccines and gene therapy. Adv. Drug Deliv. Rev. 188, 114416. 10.1016/j.addr.2022.114416 35787388 PMC9250827

[B28] Hashiba A. Toyooka M. Sato Y. Maeki M. Tokeshi M. Harashima H. (2020). The use of design of experiments with multiple responses to determine optimal formulations for *in vivo* hepatic mRNA delivery. J. Control Release 327, 467–476. 10.1016/j.jconrel.2020.08.031 32853732

[B29] Hashiba K. Sato Y. Taguchi M. Sakamoto S. Otsu A. Maeda Y. (2022). Branching ionizable lipids can enhance the stability, fusogenicity, and functional delivery of mRNA. Small sciense 3. 10.1002/smsc.202200071 PMC11935957 40212983

[B30] Hassett K. J. Higgins J. Woods A. Levy B. Xia Y. Hsiao C. J. (2021). Impact of lipid nanoparticle size on mRNA vaccine immunogenicity. J. Control Release 335, 237–246. 10.1016/j.jconrel.2021.05.021 34019945

[B31] Hatit M. Z. C. Dobrowolski C. N. Lokugamage M. P. Loughrey D. Ni H. Zurla C. (2023). Nanoparticle stereochemistry-dependent endocytic processing improves *in vivo* mRNA delivery. Nat. Chem. 15, 508–515. 10.1038/s41557-023-01138-9 36864143 PMC11831600

[B32] Hou X. Zaks T. Langer R. Dong Y. (2021). Lipid nanoparticles for mRNA delivery. Nat. Rev. Mater 6, 1078–1094. 10.1038/s41578-021-00358-0 34394960 PMC8353930

[B33] Hou X. Zhang X. Zhao W. Zeng C. Deng B. McComb D. W. (2020a). Vitamin lipid nanoparticles enable adoptive macrophage transfer for the treatment of multidrug-resistant bacterial sepsis. Nat. Nanotechnol. 15, 41–46. 10.1038/s41565-019-0600-1 31907443 PMC7181370

[B34] Hou X. Zhang X. Zhao W. Zeng C. Deng B. McComb D. W. (2020b). Author Correction: vitamin lipid nanoparticles enable adoptive macrophage transfer for the treatment of multidrug-resistant bacterial sepsis. Nat. Nanotechnol. 15, 615. 10.1038/s41565-020-0675-8 PMC10072001 32346117

[B35] Hou Y. Lin S. Xia J. Zhang Y. Yin Y. Huang M. (2023). Alleviation of ischemia-reperfusion induced renal injury by chemically modified SOD2 mRNA delivered via lipid nanoparticles. Mol. Ther. Nucleic Acids 34, 102067. 10.1016/j.omtn.2023.102067 38028193 PMC10652142

[B36] Hu C. Bai Y. Liu J. Wang Y. He Q. Zhang X. (2024). Research progress on the quality control of mRNA vaccines. Expert Rev. Vaccines 23, 570–583. 10.1080/14760584.2024.2354251 38733272

[B37] Huo H. Cheng X. Xu J. Lin J. Chen N. Lu X. (2023). A fluorinated ionizable lipid improves the mRNA delivery efficiency of lipid nanoparticles. J. Mater Chem. B 11, 4171–4180. 10.1039/d3tb00516j 37129135

[B38] Huysmans H. Zhong Z. De Temmerman J. Mui B. L. Tam Y. K. Mc Cafferty S. (2019). Expression kinetics and innate immune response after electroporation and LNP-mediated delivery of a self-amplifying mRNA in the skin. Mol. Ther. Nucleic Acids 17, 867–878. 10.1016/j.omtn.2019.08.001 31472371 PMC6722285

[B39] Johnson L. T. Zhang D. Zhou K. Lee S. M. Liu S. Dilliard S. A. (2022). Lipid nanoparticle (LNP) chemistry can endow unique *in vivo* RNA delivery fates within the liver that alter therapeutic outcomes in a cancer model. Mol. Pharm. 19, 3973–3986. 10.1021/acs.molpharmaceut.2c00442 36154076 PMC9888001

[B40] Kauffman K. J. Oberli M. A. Dorkin J. R. Hurtado J. E. Kaczmarek J. C. Bhadani S. (2018). Rapid, single-cell analysis and discovery of vectored mRNA transfection *in vivo* with a loxP-flanked tdTomato reporter mouse. Mol. Ther. Nucleic Acids 10, 55–63. 10.1016/j.omtn.2017.11.005 29499956 PMC5734870

[B41] Kawaguchi M. Noda M. Ono A. Kamiya M. Matsumoto M. Tsurumaru M. (2023). Effect of cholesterol content of lipid composition in mRNA-LNPs on the protein expression in the injected site and liver after local administration in mice. J. Pharm. Sci. 112, 1401–1410. 10.1016/j.xphs.2022.12.026 36596392 PMC9805379

[B42] Kedmi R. Ben-Arie N. Peer D. (2010). The systemic toxicity of positively charged lipid nanoparticles and the role of Toll-like receptor 4 in immune activation. Biomaterials 31, 6867–6875. 10.1016/j.biomaterials.2010.05.027 20541799

[B43] Kim M. Jeong M. Lee G. Lee Y. Park J. Jung H. (2023). Novel piperazine-based ionizable lipid nanoparticles allow the repeated dose of mRNA to fibrotic lungs with improved potency and safety. Bioeng. Transl. Med. 8, e10556. 10.1002/btm2.10556 38023699 PMC10658549

[B44] Kim Y. Choi J. Kim E. H. Park W. Jang H. Jang Y. (2024). Design of PD-L1-targeted lipid nanoparticles to turn on PTEN for efficient cancer therapy. Adv. Sci. (Weinh) 11, e2309917. 10.1002/advs.202309917 38520717 PMC11165541

[B45] Kirshina A. K. A. Kolosova E. Imasheva E. Vasileva O. Zaborova O. Terenin I. (2022). Effects of various mRNA-LNP vaccine doses on neuroinflammation in BALB/c mice. RSMU 6. 10.24075/vrgmu.2022.068

[B46] Korzun T. Moses A. S. Jozic A. Grigoriev V. Newton S. Kim J. (2024). Lipid nanoparticles elicit reactogenicity and sickness behavior in mice via toll-like receptor 4 and myeloid differentiation protein 88 Axis. ACS Nano 18, 24842–24859. 10.1021/acsnano.4c05088 39186628 PMC11916992

[B47] Kulkarni J. A. Witzigmann D. Chen S. Cullis P. R. van der Meel R. (2019). Lipid nanoparticle technology for clinical translation of siRNA therapeutics. Acc. Chem. Res. 52, 2435–2444. 10.1021/acs.accounts.9b00368 31397996

[B48] Kumar M. Kulkarni P. Liu S. Chemuturi N. Shah D. K. (2023). Nanoparticle biodistribution coefficients: a quantitative approach for understanding the tissue distribution of nanoparticles. Adv. Drug Deliv. Rev. 194, 114708. 10.1016/j.addr.2023.114708 36682420

[B49] Labonia M. Senti M. Van Der Kraak P. Brans M. Deshantri A. Dokter I. (2023). Effective cardiac mRNA delivery using lipid nanoparticles. Eur. Heart J. 44. 10.1093/eurheartj/ehad655.3301

[B50] Lam K. Schreiner P. Leung A. Stainton P. Reid S. Yaworski E. (2023). Optimizing lipid nanoparticles for delivery in primates. Adv. Mater 35, e2211420. 10.1002/adma.202211420 36972555

[B51] Lee D. Y. Amirthalingam S. Lee C. Rajendran A. K. Ahn Y. H. Hwang N. S. (2023). Strategies for targeted gene delivery using lipid nanoparticles and cell-derived nanovesicles. Nanoscale Adv. 5, 3834–3856. 10.1039/d3na00198a 37496613 PMC10368001

[B52] Lee S. Cheng Q. Yu X. Liu S. Johnson L. Siegwart D. J. (2020). A systematic study of unsaturation in lipid nanoparticles leads to improved mRNA transfection *in vivo* . Angew. Chem. 113, 5848–5853. 10.1002/anie.202013927 PMC8100975 33305471

[B53] Lei J. Qi S. Yu X. Gao X. Yang K. Zhang X. (2024). Development of mannosylated lipid nanoparticles for mRNA cancer vaccine with high antigen presentation efficiency and immunomodulatory capability. Angew. Chem. Int. Ed. Engl. 63, e202318515. 10.1002/anie.202318515 38320193

[B54] Li B. Manan R. S. Liang S. Q. Gordon A. Jiang A. Varley A. (2023). Combinatorial design of nanoparticles for pulmonary mRNA delivery and genome editing. Nat. Biotechnol. 41, 1410–1415. 10.1038/s41587-023-01679-x 36997680 PMC10544676

[B55] Li M. Li S. Huang Y. Chen H. Zhang S. Zhang Z. (2021). Secreted expression of mRNA-encoded truncated ACE2 variants for SARS-CoV-2 via lipid-like nanoassemblies. Adv. Mater 33, e2101707. 10.1002/adma.202101707 34278613 PMC8420471

[B56] Li Q. Zhang L. Lang J. Tan Z. Feng Q. Zhu F. (2023). Lipid-Peptide-mRNA nanoparticles augment radioiodine uptake in anaplastic thyroid cancer. Adv. Sci. (Weinh) 10, e2204334. 10.1002/advs.202204334 36453580 PMC9875617

[B57] Liang C. Guo B. Wu H. Shao N. Li D. Liu J. (2015). Aptamer-functionalized lipid nanoparticles targeting osteoblasts as a novel RNA interference-based bone anabolic strategy. Nat. Med. 21, 288–294. 10.1038/nm.3791 25665179 PMC5508976

[B58] Lin Y. Cheng Q. Wei T. (2023). Surface engineering of lipid nanoparticles: targeted nucleic acid delivery and beyond. Biophys. Rep. 9, 255–278. 10.52601/bpr.2023.230022 38516300 PMC10951480

[B59] Liu J. Obando D. Liao V. Lifa T. Codd R. (2011). The many faces of the adamantyl group in drug design. Eur. J. Med. Chem. 46, 1949–1963. 10.1016/j.ejmech.2011.01.047 21354674

[B60] Liu J. Q. Zhang C. Zhang X. Yan J. Zeng C. Talebian F. (2022). Intratumoral delivery of IL-12 and IL-27 mRNA using lipid nanoparticles for cancer immunotherapy. J. Control Release 345, 306–313. 10.1016/j.jconrel.2022.03.021 35301053 PMC9133152

[B61] Liu S. Cheng Q. Wei T. Yu X. Johnson L. T. Farbiak L. (2021). Membrane-destabilizing ionizable phospholipids for organ-selective mRNA delivery and CRISPR-Cas gene editing. Nat. Mater 20, 701–710. 10.1038/s41563-020-00886-0 33542471 PMC8188687

[B62] Long J. Yu C. Zhang H. Cao Y. Sang Y. Lu H. (2023). Novel ionizable lipid nanoparticles for SARS-CoV-2 omicron mRNA delivery. Adv. Healthc. Mater 12, e2202590. 10.1002/adhm.202202590 36716702 PMC11468017

[B63] LoPresti S. T. Arral M. L. Chaudhary N. Whitehead K. A. (2022). The replacement of helper lipids with charged alternatives in lipid nanoparticles facilitates targeted mRNA delivery to the spleen and lungs. J. Control Release 345, 819–831. 10.1016/j.jconrel.2022.03.046 35346768 PMC9447088

[B64] Luozhong S. Yuan Z. Sarmiento T. Chen Y. Gu W. McCurdy C. (2022). Phosphatidylserine lipid nanoparticles promote systemic RNA delivery to secondary lymphoid organs. Nano Lett. 22, 8304–8311. 10.1021/acs.nanolett.2c03234 36194390 PMC12126148

[B65] Ma F. Yang L. Sun Z. Chen J. Rui X. Glass Z. (2020). Neurotransmitter-derived lipidoids (NT-lipidoids) for enhanced brain delivery through intravenous injection. Sci. Adv. 6, eabb4429. 10.1126/sciadv.abb4429 32832671 PMC7439549

[B66] Maharjan R. Hada S. Lee J. E. Han H. K. Kim K. H. Seo H. J. (2023). Comparative study of lipid nanoparticle-based mRNA vaccine bioprocess with machine learning and combinatorial artificial neural network-design of experiment approach. Int. J. Pharm. 640, 123012. 10.1016/j.ijpharm.2023.123012 37142140

[B67] Marques A. C. Costa P. C. Velho S. Amaral M. H. (2023). Lipid nanoparticles functionalized with antibodies for anticancer drug therapy. Pharmaceutics 15, 216. 10.3390/pharmaceutics15010216 36678845 PMC9864942

[B68] Massaro M. Wu S. Baudo G. Liu H. Collum S. Lee H. (2023). Lipid nanoparticle-mediated mRNA delivery in lung fibrosis. Eur. J. Pharm. Sci. 183, 106370. 10.1016/j.ejps.2023.106370 36642345 PMC10898324

[B69] Melamed J. R. Yerneni S. S. Arral M. L. LoPresti S. T. Chaudhary N. Sehrawat A. (2023). Ionizable lipid nanoparticles deliver mRNA to pancreatic β cells via macrophage-mediated gene transfer. Sci. Adv. 9, eade1444. 10.1126/sciadv.ade1444 36706177 PMC9882987

[B70] Meyer R. A. Hussmann G. P. Peterson N. C. Santos J. L. Tuesca A. D. (2022). A scalable and robust cationic lipid/polymer hybrid nanoparticle platform for mRNA delivery. Int. J. Pharm. 611, 121314. 10.1016/j.ijpharm.2021.121314 34838950

[B71] Miao L. Li L. Huang Y. Delcassian D. Chahal J. Han J. (2019). Delivery of mRNA vaccines with heterocyclic lipids increases anti-tumor efficacy by STING-mediated immune cell activation. Nat. Biotechnol. 37, 1174–1185. 10.1038/s41587-019-0247-3 31570898

[B72] Muslimov A. Tereshchenko V. Shevyrev D. Rogova A. Lepik K. Reshetnikov V. (2023). The dual role of the innate immune system in the effectiveness of mRNA therapeutics. Int. J. Mol. Sci. 24, 14820. 10.3390/ijms241914820 37834268 PMC10573212

[B73] Ndeupen S. Qin Z. Jacobsen S. Bouteau A. Estanbouli H. Igyártó B. Z. (2021). The mRNA-LNP platform’s lipid nanoparticle component used in preclinical vaccine studies is highly inflammatory. iScience 24, 103479. 10.1016/j.isci.2021.103479 34841223 PMC8604799

[B74] Ni H. Hatit M. Z. C. Zhao K. Loughrey D. Lokugamage M. P. Peck H. E. (2022). Piperazine-derived lipid nanoparticles deliver mRNA to immune cells *in vivo* . Nat. Commun. 13, 4766. 10.1038/s41467-022-32281-5 35970837 PMC9376583

[B75] Nogueira S. S. Schlegel A. Maxeiner K. Weber B. Barz M. Schroer M. A. (2020). Polysarcosine-functionalized lipid nanoparticles for therapeutic mRNA delivery. ACS Appl. Nano Mater 11, 10634–10645. 10.1021/acsanm.0c01834

[B76] Pardi N. Tuyishime S. Muramatsu H. Kariko K. Mui B. L. Tam Y. K. (2015). Expression kinetics of nucleoside-modified mRNA delivered in lipid nanoparticles to mice by various routes. J. Control Release 217, 345–351. 10.1016/j.jconrel.2015.08.007 26264835 PMC4624045

[B77] Pardridge W. M. (2023). Brain gene therapy with Trojan horse lipid nanoparticles. Trends Mol. Med. 29, 343–353. 10.1016/j.molmed.2023.02.004 36907687 PMC10005896

[B78] Pateev I. Seregina K. Ivanov R. Reshetnikov V. (2023). Biodistribution of RNA vaccines and of their products: evidence from human and animal studies. Biomedicines 12, 59. 10.3390/biomedicines12010059 38255166 PMC10812935

[B79] Patel S. Ryals R. C. Weller K. K. Pennesi M. E. Sahay G. (2019). Lipid nanoparticles for delivery of messenger RNA to the back of the eye. J. Control Release 303, 91–100. 10.1016/j.jconrel.2019.04.015 30986436 PMC6579630

[B80] Pattipeiluhu R. Arias-Alpizar G. Basha G. Chan K. Y. T. Bussmann J. Sharp T. H. (2022). Anionic lipid nanoparticles preferentially deliver mRNA to the hepatic reticuloendothelial system. Adv. Mater 34, e2201095. 10.1002/adma.202201095 35218106 PMC9461706

[B81] Paunovska K. Da Silva Sanchez A. J. Lokugamage M. P. Loughrey D. Echeverri E. S. Cristian A. (2022). The extent to which lipid nanoparticles require apolipoprotein E and low-density lipoprotein receptor for delivery changes with ionizable lipid structure. Nano Lett. 22, 10025–10033. 10.1021/acs.nanolett.2c03741 36521071

[B82] Paunovska K. Da Silva Sanchez A. J. Sago C. D. Gan Z. Lokugamage M. P. Islam F. Z. (2019). Nanoparticles containing oxidized cholesterol deliver mRNA to the liver microenvironment at clinically relevant doses. Adv. Mater 31, e1807748. 10.1002/adma.201807748 30748040 PMC6445717

[B83] Priya S. Desai V. M. Singhvi G. (2023). Surface modification of lipid-based nanocarriers: a potential approach to enhance targeted drug delivery. ACS Omega 8, 74–86. 10.1021/acsomega.2c05976 36643539 PMC9835629

[B84] Qiu M. Tang Y. Chen J. Muriph R. Ye Z. Huang C. (2022). Lung-selective mRNA delivery of synthetic lipid nanoparticles for the treatment of pulmonary lymphangioleiomyomatosis. Proc. Natl. Acad. Sci. U. S. A. 119, e2116271119. 10.1073/pnas.2116271119 35173043 PMC8872770

[B85] Radloff K. Gutbier B. Dunne C. M. Moradian H. Schwestka M. Gossen M. (2023). Cationic LNP-formulated mRNA expressing Tie2-agonist in the lung endothelium prevents pulmonary vascular leakage. Mol. Ther. Nucleic Acids 34, 102068. 10.1016/j.omtn.2023.102068 38034031 PMC10682670

[B86] Radmand A. Lokugamage M. P. Kim H. Dobrowolski C. Zenhausern R. Loughrey D. (2023). The transcriptional response to lung-targeting lipid nanoparticles *in vivo* . Nano Lett. 23, 993–1002. 10.1021/acs.nanolett.2c04479 36701517 PMC9912332

[B87] Ramezanpour M. Tieleman D. P. (2022). Computational insights into the role of cholesterol in inverted hexagonal phase stabilization and endosomal drug release. Langmuir 38, 7462–7471. 10.1021/acs.langmuir.2c00430 35675506 PMC9220946

[B88] Rurik J. G. Tombacz I. Yadegari A. Mendez Fernandez P. O. Shewale S. V. Li L. (2022). CAR T cells produced *in vivo* to treat cardiac injury. Science 375, 91–96. 10.1126/science.abm0594 34990237 PMC9983611

[B89] Ryals R. C. Patel S. Acosta C. McKinney M. Pennesi M. E. Sahay G. (2020). The effects of PEGylation on LNP based mRNA delivery to the eye. PLoS One 15, e0241006. 10.1371/journal.pone.0241006 33119640 PMC7595320

[B90] Sarode A. Fan Y. Byrnes A. E. Hammel M. Hura G. L. Fu Y. (2022). Predictive high-throughput screening of PEGylated lipids in oligonucleotide-loaded lipid nanoparticles for neuronal gene silencing. Nanoscale Adv. 4, 2107–2123. 10.1039/d1na00712b 36133441 PMC9417559

[B91] Sato Y. (2021). Development of lipid nanoparticles for the delivery of macromolecules based on the molecular design of pH-sensitive cationic lipids. Chem. Pharm. Bull. (Tokyo) 69, 1141–1159. 10.1248/cpb.c21-00705 34853281

[B92] Schellekens R. C. Stellaard F. Woerdenbag H. J. Frijlink H. W. Kosterink J. G. (2011). Applications of stable isotopes in clinical pharmacology. Br. J. Clin. Pharmacol. 72, 879–897. 10.1111/j.1365-2125.2011.04071.x 21801197 PMC3244634

[B93] Sedic M. Senn J. J. Lynn A. Laska M. Smith M. Platz S. J. (2018). Safety evaluation of lipid nanoparticle-formulated modified mRNA in the sprague-dawley rat and cynomolgus monkey. Vet. Pathol. 55, 341–354. 10.1177/0300985817738095 29191134

[B94] Shen Z. Liu C. Wang Z. Xie F. Liu X. Dong L. (2023). Development of a library of disulfide bond-containing cationic lipids for mRNA delivery. Pharmaceutics 15, 477. 10.3390/pharmaceutics15020477 36839799 PMC9961079

[B95] Shimosakai R. Khalil I. A. Kimura S. Harashima H. (2022). mRNA-Loaded lipid nanoparticles targeting immune cells in the spleen for use as cancer vaccines. Pharm. (Basel) 15, 1017. 10.3390/ph15081017 PMC9415712 36015165

[B96] Shiraishi K. Yokoyama M. (2019). Toxicity and immunogenicity concerns related to PEGylated-micelle carrier systems: a review. Sci. Technol. Adv. Mater 20, 324–336. 10.1080/14686996.2019.1590126 31068982 PMC6493319

[B97] Swingle K. L. Safford H. C. Geisler H. C. Hamilton A. G. Thatte A. S. Billingsley M. M. (2023). Ionizable lipid nanoparticles for *in vivo* mRNA delivery to the placenta during pregnancy. J. Am. Chem. Soc. 145, 4691–4706. 10.1021/jacs.2c12893 36789893 PMC9992266

[B98] Tenchov R. Bird R. Curtze A. E. Zhou Q. (2021). Lipid Nanoparticles─From liposomes to mRNA vaccine delivery, a landscape of research diversity and advancement. ACS Nano 15, 16982–17015. 10.1021/acsnano.1c04996 34181394

[B99] Tilstra G. Couture-Senecal J. Lau Y. M. A. Manning A. M. Wong D. S. M. Janaeska W. W. (2023). Iterative design of ionizable lipids for intramuscular mRNA delivery. J. Am. Chem. Soc. 145, 2294–2304. 10.1021/jacs.2c10670 36652629

[B100] Tombacz I. Laczko D. Shahnawaz H. Muramatsu H. Natesan A. Yadegari A. (2021). Highly efficient CD4+ T cell targeting and genetic recombination using engineered CD4+ cell-homing mRNA-LNPs. Mol. Ther. 29, 3293–3304. 10.1016/j.ymthe.2021.06.004 34091054 PMC8571164

[B101] Tuma J. Chen Y. J. Collins M. G. Paul A. Li J. Han H. (2023). Lipid nanoparticles deliver mRNA to the brain after an intracerebral injection. Biochemistry 62, 3533–3547. 10.1021/acs.biochem.3c00371 37729550 PMC10760911

[B102] Wadhera R. K. Steen D. L. Khan I. Giugliano R. P. Foody J. M. (2016). A review of low-density lipoprotein cholesterol, treatment strategies, and its impact on cardiovascular disease morbidity and mortality. J. Clin. Lipidol. 10, 472–489. 10.1016/j.jacl.2015.11.010 27206934

[B103] Waggoner L. E. Miyasaki K. F. Kwon E. J. (2023). Analysis of PEG-lipid anchor length on lipid nanoparticle pharmacokinetics and activity in a mouse model of traumatic brain injury. Biomater. Sci. 11, 4238–4253. 10.1039/d2bm01846b 36987922 PMC10262813

[B104] Xiong H. Liu S. Wei T. Cheng Q. Siegwart D. J. (2020). Theranostic dendrimer-based lipid nanoparticles containing PEGylated BODIPY dyes for tumor imaging and systemic mRNA delivery *in vivo* . J. Control Release 325, 198–205. 10.1016/j.jconrel.2020.06.030 32629133 PMC8152631

[B105] Xu Y. Hu Y. Xia H. Zhang S. Lei H. Yan B. (2024). Delivery of mRNA vaccine with 1, 2-diesters-derived lipids elicits fast liver clearance for safe and effective cancer immunotherapy. Adv. Healthc. Mater 13, e2302691. 10.1002/adhm.202302691 37990414

[B106] Yang T. Li C. Wang X. Zhao D. Zhang M. Cao H. (2020). Efficient hepatic delivery and protein expression enabled by optimized mRNA and ionizable lipid nanoparticle. Bioact. Mater 5, 1053–1061. 10.1016/j.bioactmat.2020.07.003 32691013 PMC7355334

[B107] Young R. E. Nelson K. M. Hofbauer S. I. Vijayakumar T. Alameh M. G. Weissman D. (2022). Lipid nanoparticle composition drives mRNA delivery to the placenta. bioRxiv. 10.1101/2022.12.22.521490

[B108] Yu X. Liu S. Cheng Q. Wei T. Lee S. Zhang D. (2020). Lipid-modified aminoglycosides for mRNA delivery to the liver. Adv. Healthc. Mater 9, e1901487. 10.1002/adhm.201901487 32108440 PMC8152636

[B109] Yu X. Xu T. Shi H. Hong J. Jin X. Cao L. (2023). Cartilage-targeting mRNA-lipid nanoparticles rescue perifocal apoptotic chondrocytes for integrative cartilage repair. Chem. Eng. J. 465, 142841. 10.1016/j.cej.2023.142841

[B110] Zhang H. Leal J. Soto M. R. Smyth H. D. C. Ghosh D. (2020). Aerosolizable lipid nanoparticles for pulmonary delivery of mRNA through design of experiments. Pharmaceutics 12, 1042. 10.3390/pharmaceutics12111042 33143328 PMC7692784

[B111] Zhou J. E. Sun L. Jia Y. Wang Z. Luo T. Tan J. (2022). Lipid nanoparticles produce chimeric antigen receptor T cells with interleukin-6 knockdown *in vivo* . J. Control Release 350, 298–307. 10.1016/j.jconrel.2022.08.033 36002054

